# The Vitrectomy Timing Individualization System for Ocular Trauma (VTISOT)

**DOI:** 10.1038/s41598-019-48862-2

**Published:** 2019-08-30

**Authors:** Longhui Han, Jinchen Jia, Yiming Fan, Luyong Yang, Zhiqiang Yue, Wei Zhang, Fang Liu, Huanjun Kang, Tao Huo, Shaolei Han, Hua Shen, Genquan Tian, Xuemin Su

**Affiliations:** 1 Hebei Provincial Eye institute, Hebei Provincial Eye Hospital, Xingtai, Hebei 054001 China; 2 Tianjin Medical University Eye Hospital/Eye Institute, School of Optometry and Ophthalmology, Tianjin Medical University, Tianjin, 300384 China; 3 North China University of Science and Technology, Tangshan, Hebei 063210 China

**Keywords:** Reconstruction, Outcomes research, Risk factors

## Abstract

Ocular trauma is a major cause of monocular blindness worldwide. Vitrectomy at correct timing can significantly improve the efficacy and prognosis, but the timing of vitrectomy has remained highly controversial for decades. Trauma cases are different from each other, thus, a flexible timing system based on the details of each individual case is recommended. Unfortunately, no such a timing system is available for clinical application up to now. To establish the vitrectomy timing individualization system for ocular trauma (VTISOT), we first identified 6 independent tPVR risk factors (including Zone 3 Injury, Zone 3 retinal Laceration, Massive Vitreous Hemorrhage, Retinal Disorder, Timing of Vitrectomy and Type of Injury) by retrospective study. Then, the tPVR score was established by binary logistic regression analysis. Most importantly and critically, the vitrectomy timing individualization system for ocular trauma was established based on the identified tPVR risk factors and the tPVR score. The following evaluation of the VTISOT showed that the patients consistent with the VTISOT principles exhibited reduced tPVR incidence and better surgical results. In short, the VTISOT principles were established, which may provide a new approach to individualize the timing of vitrectomy and improve the prognosis after trauma.

## Introduction

Ocular trauma is a major cause of monocular blindness worldwide. The prognosis for a serious eye injury is primarily determined by the initial injury, the quality and timing of surgical intervention, and the biological reaction of the body^1^. Among these factors, there is obviously no way of reducing the damage inflicted by the initial injury. Fortunately, with the introduction and development of vitrectomy, the quality of surgical reconstruction has substantially improved. However, unfortunately, the scarformation reaction of the body, described as traumatic proliferative vitreoretinopathy (tPVR) in the posterior segment, has remained a crucial limiting factor in the rehabilitation of the severely injured eye^1^.

Vitrectomy at the correct timing not only reduces the incidence of tPVR but also significantly improves the efficacy and prognosis. But the timing of vitrectomy has remained highly controversial for decades^1–5^. The main reason for this long-standing controversy is that trauma cases are different from each other, and it is impossible for us to provide a unified surgical timing for various eye injuries. Thus, a flexible approach based on the details of the individual case is recommended^4,6^. Unfortunately, no vitrectomy timing individualization system is available for clinical application up to now.

In this study, we established the vitrectomy timing individualization system for ocular trauma (VTISOT) on the basis of tPVR risk factors and the tPVR score, providing a new method to individualize the timing of vitrectomy and improve the prognosis for ocular trauma.

## Methods

### Ethics, patients and study design

This retrospective study was approved by the institutional review and ethics boards of the Hebei Provincial Eye Hospital, Hebei, China and followed the tenets of the Declaration of Helsinki. All research was performed in accordance with relevant guidelines and regulations. Informed written consent was obtained from all the parents.

We enrolled consecutive patients who were evaluated at the ocular trauma department of the Hebei Provincial Eye Hospital between Jan 2014 and Nov 2017. Their medical records were reviewed for the following data: age, sex, time of injury, type of injury (penetrating, rupture, contusion or perforating)^7^, injured eye (OD or OS), preoperative best-corrected visual acuity (BCVA) and intraocular pressure (IOP), cornea (clear or injured), hyphema (grades 0~4)^8^, lens (injured or uninjured), length of laceration, zone of wound^7^, retinal condition, vitreous hemorrhage (VH), timing of surgery, intra-operative major hemorrhage, iatrogenic retinal injury, and postoperative tPVR, BCVA, retinal detachment (RD), infection, and globe survival (survival, phthisis, evisceration or enucleation).

The inclusion criteria were as follows: (1) patients diagnosed with eye injury; (2) those who underwent pars plana vitrectomy. The exclusion criteria were as follows: (1) patients who were injured for more than 10 days at the initial presentation; (2) a history of eye injury; (3) those in which endophthalmitis or intraocular foreign body was diagnosed at the initial presentation; (4) those who underwent early endoscopic vitrectomy due to severe corneal edema and opacity; (5) those who underwent postponed pars plana vitrectomy due to severe corneal edema and opacity; (6) massive choroidal hemorrhage diagnosed by preoperative ultra B-scan; (7) those who underwent only anterior vitrectomy; (8) incomplete medical records; (9) those with a follow-up period <6 months after vitrectomy.

The tPVR risk factors were identified by univariate and multivariate analyses. The tPVR score was established by binary logistic regression analysis according to the identified tPVR risk factors. Then, the vitrectomy timing individualization system for ocular trauma (VTISOT) was established based on the tPVR risk factors, the tPVR score and the vitrectomy-associated characteristics of the injured eyes.

To verify the validity of the VTISOT principles, we divided the patients who underwent vitrectomy into two groups according to the consistency between the actual timing of vitrectomy and the individualized timing from VTISOT: Consistency Group and Inconsistency Group. Then, we compared the differences in tPVR, postoperative RD, globe survival, intra-operative major hemorrhage, iatrogenic retinal injury, postoperative infection, postoperative BCVA and BCVA improvement between the two groups.

### Definition of tPVR

According to Pastor JC, etc., PVR is defined as the growth and contraction of cellular membranes within the vitreous cavity and on both retinal surfaces, and in many cases a fibrotic process of the retina itself^9,10^. Thus, the tPVR in our study was defined as the cellular membranes within the vitreous cavity or on either retinal surfaces or obvious retinal shrinkage after trauma.

### Massive vitreous hemorrhage

A red reflex is present with no retinal detail observed posterior to the equator or no red reflex or the same amount of blood was observed during vitrectomy. The hemorrhage is equivalent to grades 3 and 4 in T Göncü’s vitreous hemorrhage grading^11^.

### Zones of mechanical eyeglobe injury

Open-globe injury: Zone 1: Isolated to cornea (including the corneoscleral limbus); Zone 2: Corneoscleral limbus to a point 5 mm posterior into the sclera; Zone 3: Posterior to the anterior 5 mm of sclera. Closed-globe injury: Zone 1: external (limited to bulbar conjunctiva, sclera, and cornea); Zone 2: anterior segment (involving structures in anterior segment internal to the cornea and including the posterior lens capsule; also includes pars plicata but not pars plana); Zone 3: posterior segment (all internal structures posterior to the posterior lens capsule)^7^.

### Zone 3 retinal (Z3R) laceration

Retinal laceration appears but sclera does not have a full-thickness wound in zone 3 of the open-globe eye injury, for example, a corneal penetrating eye injury by a steel nail with retinal laceration in zone 3.

### Retinal disorder

A portion of or the entire retina cannot be recognized by B-ultrasonography. In other words, the vitreoretinal interface is seriously blurred.

### Timing of vitrectomy

Based on F Kuhn’s review, the timing of vitrectomy in our research includes the following options: early (days 2–4), delayed (days 5–7), late (days 8–14), and very late (past 2 weeks). The timing refers to the interval of injury and vitrectomy measured in days^4^.

### Grade of visual acuity (VA)

Based on the grading method of DJ Pieramici, the VA was divided into the following 7 grades: no light perception (NLP), light perception (LP), hand motion (HM), counting finger (CF) ~ 4/200, 5/200~19/100, 20/100~20/50, and ≥20/40^7^.

### Hyphema grading

Grade 0: without blood; Grade 1, hyphema filling less than one-third of the anterior chamber (AC); grade 2, hyphema filling one-third to one-half of the AC; grade 3, hyphema filling more than half of the AC but less than the total; and grade 4, total hyphema with either red or black blood clots^8,12^.

### Statistical analysis

Differences in BCVA and BCVA improvement between groups were assessed using the Mann-Whitney U test. Differences in age and length of laceration between groups were assessed using Student’s t-test. Differences in other characteristics between groups were assessed by Fisher’s exact test or the Chi-Square test. Multivariable analyses for tPVR risk factors were performed by binary logistic regression analysis. To determine the cutoff value of the tPVR score, ROC curve analysis was applied. All analyses were performed using SPSS software ver. 21.0 (SPSS Inc, Chicago, IL). The level of significance was set at *P* < 0.05.

## Results

### Demographics and ocular characteristics of the study

A total of 625 consecutive injured eyes that met the initial inclusion criteria were evaluated. Of these, 320 eyes were excluded because of exclusion criteria. A total of 305 eyes from 305 injured patients were included in our final analysis. Of the 305 included eyes, 137 (49.9%) eyes had no tPVR, and 168 (55.1%) eyes had tPVR after vitrectomy. The demographics and ocular characteristics of the 2 groups (no tPVR group and tPVR group) are summarized (Tables 1–3).

**Table 1:** Univariable statistics for tPVR: Fisher’s Exact Test or Chi-Square Test.

Characteristics	Subgroups	tPVR (0), N (%)	tPVR (1), N (%)	*Х* ^2^	*P* Value
Total		137 (49.9)	168 (55.1)		
Sex	Male	120 (44.8)	148 (55.2)	0.018	1.000
	Female	17 (45.9)	20 (54.1)		
Injured Eye	OD	65 (43.3)	85 (56.7)	0.300	0.645
	OS	72 (46.5)	83 (53.5)		
Zone 3 Injury	0	61 (59.8)	41 (40.2)	13.680	<0.001*
	1	76 (37.4)	127 (62.6)		
Z3R Laceration	0	132 (46.6)	151 (53.4)	4.704	0.043*
	1	5 (22.7)	17 (77.3)		
Massive VH	0	109 (52.4)	99 (47.6)	14.764	<0.001*
	1	28 (28.9)	69 (71.1)		
Retinal Disorder	0	99 (58.6)	70 (41.4)	28.498	<0.001*
	1	38 (27.9)	98 (72.1)		
Timing of Vitrectomy	Days 2–4	78 (66.7)	39 (33.3)	38.025	<0.001*
	Days 5–7	21 (38.9)	33 (61.1)		
	Days 8–14	35 (28.2)	89 (71.8)		
	Past 2 Weeks	3 (30.0)	7 (70.0)		
Type of Injury	Penetrating	55 (57.3)	41 (42.7)	11.078	0.011*
	Rupture	48 (42.5)	65 (57.5)		
	Contusion	33 (37.1)	56 (62.9)		
	Perforating	1 (14.3)	6 (85.7)		
Corneal Injury	0	51 (41.5)	72 (58.5)	0.991	0.349
	1	86 (47.3)	96 (52.7)		
Hyphema	Grade 0	74 (42.8)	99 (57.2)	2.025	0.731
	Grade 1	44 (47.3)	49 (52.7)		
	Grade 2	2 (40.0)	3 (60.0)		
	Grade 3	6 (40.0)	9 (60.0)		
	Grade 4	11 (57.9)	8 (42.1)		
IOP (Pre-Op)	<11 mmHg	29 (45.3)	35 (54.7)	3.125	0.210
	11–21 mmHg	85 (42.3)	116 (57.7)		
	>21 mmHg	23 (57.5)	17 (42.5)		
Lens’ Injury	0	27 (45.8)	32 (54.2)	0.885	0.499
	1	110 (44.7)	136 (55.3)		

tPVR: traumatic proliferative vitreoretinopathy; VH: vitreous hemorrhage; IOP: intraocular pressure; Pre-Op: pre-operative. 0: no; 1: yes.

Z3R Laceration: retinal laceration appears but sclera does not have a full-thickness wound in zone 3 (posterior to the anterior 5 mm of sclera) of the open globe eye injury, for example, a corneal penetrating eye injury by a steel nail with retinal laceration in zone 3.

^*^Represent *P* Value < 0.05.

**Table 2:** Univariable statistics for tPVR: Rank-Sum Test (Mann-Whitney U Test).

Characteristic	Subgroups	N (%)	Mean Rank	Z	*P* Value
BCVA (Pre-Op)	tPVR (0)	137 (49.9)	154.91	−0.363	0.716
	tPVR (1)	168 (55.1)	151.44		

tPVR: traumatic proliferative vitreoretinopathy; BCVA: best corrected visual acuity; Pre-Op: pre-operative; 0: no; 1: yes.

**Table 3:** Univariable statistics for tPVR: T-Test.

Characteristics	Subgroups	N (%)	Mean (SD)	*t*	*P* Value
Age (years)	tPVR (0)	137 (49.9)	42.56 (17.13)	−0.421	0.674
	tPVR (1)	168 (55.1)	43.34 (15.06)		
Length of Laceration	tPVR (0)	137 (49.9)	10.60 (8.40)	0.643	0.521
	tPVR (1)	168 (55.1)	9.93 (9.54)		

tPVR: traumatic proliferative vitreoretinopathy; 0: no; 1: yes.

### Identification of the tPVR risk factors

To identify tPVR risk factors, we first processed univariable statistics. The results revealed significant differences in Zone 3 Injury, Z3R Laceration, Massive VH, Retinal Disorder, Timing of Vitrectomy and Type of Injury between the no tPVR group and tPVR group (Tables 1–3). No significant differences in Sex, Eye difference, Age and other characteristics of the injured eyes, including Corneal Injury, Hyphema, Preoperative IOP, Lens’ Injury, Preoperative BCVA and Length of Laceration, were noted. The following multivariable analyses revealed results similar to that of the univariable analyses (Table 4). According to the above results, we identified Zone 3 Injury, Z3R Laceration, Massive VH, Retinal Disorder, Timing of Vitrectomy and Type of Injury as tPVR risk factors.

**Table 4:** Multivariable ORs for tPVR: Logistic Regression Analysis.

Characteristics	Subgroups	Patients, N (%)	OR	95% CI		*P* Value
				Lower	Upper	
All		305 (100)				
Zone 3 Injury	0	102 (33.44)	1			
	1	203 (66.56)	18.210	7.358	45.067	<0.001*
Z3R Laceration	0	283 (92.79)	1			
	1	22 (7.21)	28.774	5.451	151.883	<0.001*
Massive VH	0	208 (68.20)	1			
	1	97 (31.80)	5.391	2.172	13.379	<0.001*
Retinal Disorder	0	169 (55.41)	1			
	1	136 (44.59)	4.642	2.023	10.654	<0.001*
Timing of Vitrectomy	Days 2–4	117 (38.36)	1			
	Days 5–7	54 (17.70)	9.171	3.486	24.123	<0.001*
	Days 8–14	124 (40.66)	35.512	13.743	91.764	<0.001*
	Past 2 Weeks	10 (3.28)	42.036	6.052	291.984	<0.001*
Type of Injury	Penetrating	96 (31.48)	1			
	Rupture	113 (37.05)	2.891	1.086	7.694	0.034*
	Contusion	89 (29.18)	4.310	1.278	14.533	0.018*
	Perforating	7 (2.30)	42.923	2.704	681.484	0.008*
Age		305 (100)	1.000	0.979	1.022	0.990
Sex	Male	268 (87.87)	1			
	Female	37 (12.13)	0.966	0.357	2.615	0.946
Eye	OD	150 (49.18)	1			
	OS	155 (50.82)	0.692	0.354	1.354	0.283
BCVA (Pre-Op)		305 (100)	0.927	0.593	1.450	0.740
Corneal Injury	0	123 (40.33)	1			
	1	182 (59.67)	1.216	0.590	2.509	0.596
Hyphema	Grade 0	173 (56.72)	1			
	Grade 1	93 (30.49)	0.949	0.431	2.091	0.898
	Grade 2	5 (1.64)	0.271	0.016	4.514	0.363
	Grade 3	15 (4.92)	0.466	0.085	2.559	0.380
	Grade 4	19 (6.23)	1.544	0.383	6.218	0.541
IOP (Pre-Op)	<11 mmHg	64 (20.98)	1			
	11–21 mmHg	201 (65.90)	1.777	0.748	4.221	0.192
	>21 mmHg	40 (13.11)	0.991	0.294	3.336	0.988
Lens’ Injury	0	59 (19.34)	1			
	1	246 (80.66)	0.931	0.399	2.171	0.868
Length of Laceration		305 (100)	0.997	0.937	1.062	0.932

tPVR: traumatic proliferative vitreoretinopathy; OR: odds ratio; CI: confidence interval; VH: vitreous hemorrhage; BCVA: best corrected visual acuity. Pre-Op: pre-operative. IOP: intraocular pressure. 0: no; 1: yes.

Z3R Laceration: retinal laceration appears but sclera does not have a full-thickness wound in zone 3 (posterior to the anterior 5 mm of sclera) of the open globe eye injury, for example, a corneal penetrating eye injury by a steel nail with retinal laceration in zone 3.

^*^Represent *P* Value < 0.05.

We also found that the incidence of tPVR in the early surgery group (Days 2–4, 33.3%) was considerably reduced compared with other groups (Days 5–7, 61.1%; Days 8–14, 71.8%; Past 2 Weeks, 70.0%). This finding indicates that early vitrectomy may decrease the incidence of postoperative tPVR.

### Establishment of *the tPVR score*

To predict the incidence of postoperative tPVR before vitrectomy, we established the tPVR score using binary logistic regression analysis (Table 5).

**Table 5:** Variables in the equation for predicting post-operative tPVR: Binary Logistic Regression Analysis.

Variables	Subgroups	B	S.E.	*P* Value	OR	95% CI	
						Lower	Upper
Zone 3 Injury	0	0			1		
	1	1.84	0.349	<0.001*	6.297	3.18	12.468
Z3R Laceration	0	0			1		
	1	2.721	0.681	<0.001*	15.203	4.006	57.7
Massive VH	0	0			1		
	1	0.785	0.348	***0.0*** **2** 4*	2.193	1.109	4.335
Retinal Disorder	0	0			1		
	1	0.759	0.306	0.013*	2.137	1.173	3.895
Type of Injury	Penetrating	0			1		
	Rupture	0.914	0.346	0.008*	2.495	1.266	4.918
	Contusion	1.48	0.376	<0.001*	4.393	2.1	9.187
	Perforating	2.247	1.129	0.047*	9.456	1.034	86.47
Constant		−2.583	0.421	<0.001*	0.076		

tPVR: traumatic proliferative vitreoretinopathy; OR: odds ratio; CI: confidence interval; VH: vitreous hemorrhage.

Z3R Laceration: retinal laceration appears but sclera does not have a full-thickness wound in zone 3 (posterior to the anterior 5 mm of sclera) of the open globe eye injury, for example, a corneal penetrating eye injury by a steel nail with retinal laceration in zone 3.

Omnibus Tests of Model Coefficients (Model *Х*^2^ = 78.360, *P* < 0.001).

Hosmer and Lemeshow Test (*Х*^*2*^ = 12.430*, P* = 0.133 > 0.05).

Classification Table: The cut value is 0.500 (Predicted Probability), the Overall Percentage is 69.2%.

^*^Represent *P* Value < 0.05.

**The tPVR score** = Logit (P) = Zone 3 Injury × 1.84 + Z3R Laceration × 2.721 + Massive VH × 0.785 + Retinal Disorder × 0.785 + Rupture × 0.914 + Contusion × 1.48 + Perforating × 2.247 − 2.583.

**Figure 1:**
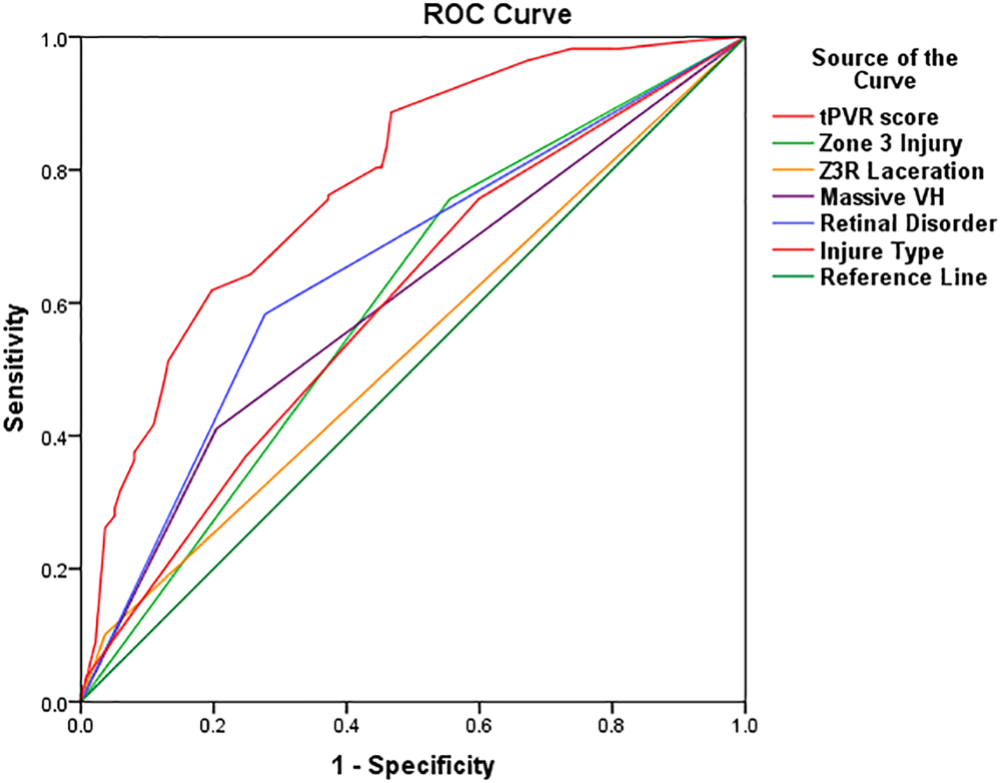
ROC Curve showed that compared with any individual tPVR risk factors, tPVR score predicted tPVR better.

ROC curves revealed that compared with any individual tPVR risk factors, tPVR score predicted tPVR better (Fig. 1 and Table 6). The cutoff value calculated based on Jordan index was 0.589, and the sensitivity and specificity are 0.619 and 0.813, respectively (Table 6). The possibility of postoperative tPVR is 64.3% when the tPVR score is 0.589. The greater the tPVR score, the higher the probability of postoperative tPVR.

$$\underline{{\bf{The}}\,{\bf{predicted}}\,{\bf{incidence}}\,{\bf{of}}\,{\bf{tPVR}}}=\frac{{e}^{{\rm{tPVR}}{\rm{score}}}}{1+{e}^{{\rm{tPVR}}{\rm{score}}}}$$

**Table 6:** ROC Curve cutoff value of the tPVR score for predicting tPVR.

	AUC (95% CI)	*S.E*.	*P*	cutoff value	Sensitivity	Specificity
tPVR score	0.783 (0.731~0.834)	0.026	<0.001	0.589	0.619	0.813

ROC Curve: receiver operation characteristic curve; tPVR: traumatic proliferative vitreoretinopathy; AUC: area under the curve; CI: confidence interval.

### *Establishment* of the vitrectomy timing individualization system for ocular trauma (VTISOT)

The timing of vitrectomy for ocular trauma is mainly based on the tPVR risk factors. The individualized adjustments are based on the tPVR score and the vitrectomy-associated characteristics of the injured eyes. The following principles apply to vitrectomy for non-endophthalmitis and non-IOFB.

1. **Four main tPVR risk factors and classification of high/low tPVR risk before vitrectomy**

1.1 **Four main tPVR risk factors:** Zone 3 Injury, Z3R Laceration, Massive VH, and Retinal Disorder.

1.2 **High tPVR risk case:** A case that includes one or more of the above 4 main tPVR risk factors.

1.3 **Low tPVR risk case:** A case that includes none of the above 4 main tPVR risk factors.

2. **Timing of PPV surgery**

2.1 **High tPVR risk cases:** Days 2–4 after injury.

2.2 **Low tPVR risk cases:** Days 8–14 after injury.

3. **Individualized adjustments of surgical timing (on the basis of not violating the above principles)**

3.1 **Adjust surgical timing by tPVR score**

3.1.1  There is a possibility of tPVR when the tPVR score is greater than 0.589. The greater the tPVR score, the higher the probability of tPVR.

3.1.2  Early surgery may reduce the incidence of tPVR. Thus, the greater the tPVR score, the earlier the surgery.

3.1.3  The surgical timing should be delayed properly when tPVR score is less than 0.589. The lower the tPVR score, the later the surgery.

3.2 **Adjust surgical timing by characteristics of the injured eye**

3.2.1 Corneal edema: The surgical timing should be delayed properly when the cornea is not clear enough due to edema. Otherwise, endoscopic vitrectomy or temporary keratoprosthesis should be applied^4^.

3.2.2  Suprachoroidal hemorrhage: The surgical timing should be delayed properly when suprachoroidal hemorrhage exits^6,13^.

3.2.3  Unsutured wounds: Unsutured wounds (too posterior to reach) generally require some delay, and the IOP should be tightly controlled during vitrectomy^4^.

3.2.4  Lens injury: Severe glaucoma second to lens injury requires the earlier vitrectomy^4^.

3.2.5  Vitreous hemorrhage: The more severe the VH, the more urgent its removal^4^.

3.2.6  Low IOP: The lower the IOP, the earlier the vitrectomy should be performed (to prevent secondary ciliary body damage and consequent phthisis)^4^.

### Evaluation of the VTISOT

To evaluate the VTISOT, we divided the patients into 2 groups: inconsistency group (164, 53.8%) and consistency group (141, 46.2%). The consistency group exhibited a reduced incidence of tPVR (27.0%), postoperative RD (9.9%), and iatrogenic retinal injury (4.3%) and increased incidence of globe survival (98.6%) compared with the inconsistency group (79.3%, 39.65%, 11.0% and 90.9%) (Table 7). The postoperative BCVA and the BCVA improvement in consistency group were improved compared with the inconsistency group (Table 8). No significant differences in intra-operative major hemorrhage and postoperative infection were noted between the consistency and inconsistency groups (Table 7). Taken together, the patients consistent with the VTISOT principles exhibited reduced tPVR incidence and better surgical results.

**Table 7:** Statistics for the evaluation of VTISOT: Fisher’s Exact Test or Chi-Square Test.

Characteristics	Subgroups	Consistency, N (%)		*Х* ^2^	*P* Value
		0	1		
Total		164 (53.8)	141 (46.2)		
tPVR	0	34 (20.7)	103 (73.0)	83.600	<0.001*
	1	130 (79.3)	38 (27.0)		
RD (Post-Op)	0	99 (60.4)	127 (90.1)	34.743	<0.001*
	1	65 (39.6)	14 (9.9)		
Globe Survival	0	15 (9.1)	2 (1.4)	8.575	0.004*
	1	149 (90.9)	139 (98.6)		
Intra-Op Major Hemorrhage	0	150 (91.5)	128 (90.8)	0.044	0.843
	1	14 (8.5)	13 (9.2)		
Iatrogenic Retinal Injury	0	146 (89.0)	135 (95.7)	4.708	0.034*
	1	18 (11.0)	6 (4.3)		
Post-operative Infection	0	163 (99.4)	141 (100)	0.860	1.000
	1	1 (0.6)	0 (0)		

VTISOT: the vitrectomy timing individualization system for ocular trauma; tPVR: traumatic proliferative vitreoretinopathy; RD: retinal detachment; Post-Op: post-operative; Intra-Op: intra-operative; 0: no; 1: yes.

Globe Survival (0): phthisis, evisceration or enucleation.

Consistency (0): The actual timing of vitrectomy is inconsistent with the timing of VTISOT.

Consistency (1): The actual timing of vitrectomy is consistent with the timing of VTISOT.

*Represent *P* Value < 0.05.

**Table 8:** Statistics for the evaluation of VTISOT: Rank-Sum Test (Mann-Whitney U Test).

Characteristic	Subgroups	N (%)	Mean Rank	Z	*P* Value
BCVA (Post-Op)	Consistency (0)	164 (53.8)	132.27	−4.580	<0.001*
	Consistency (1)	141 (46.2)	177.12		
BCVA Improvement	Consistency (0)	164 (53.8)	132.24	−4.677	<0.001*
	Consistency (1)	141 (46.2)	177.15		

VTISOT: the vitrectomy timing individualization system for ocular trauma; BCVA: best corrected visual acuity; Post-Op: post-operative.

Consistency (0): The actual timing of vitrectomy is inconsistent with the individualized timing from VTISOT.

Consistency (1): The actual timing of vitrectomy is consistent with the individualized timing from VTISOT.

*Represent *P* Value < 0.05.

## Discussion

In this study, the vitrectomy timing individualization system for ocular trauma (VTISOT) were established based on the independent tPVR risk factors and the tPVR score.

Ocular trauma is a major cause of monocular blindness worldwide. Despite progress made in surgical techniques, pharmacological adjuncts, and preventive measurements, the clinical outcome remains unsatisfactory. Especially, The timing of vitrectomy for ocular trauma has remained highly controversial for decades^1–5^. Numerous patients miss the best opportunity to regain their sight or even anatomically lose their eyeballs (phthisis, enucleation, evisceration).

The main reason for this long-standing controversy in vitrectomy timing after trauma is that no two injuries are identical and no two surgeons are the same. An individual plan must be fashioned in each case based on the specifics of the injured eye and patient and the capabilities, experience and expertise of the surgeon^4^. Issues involving logistics and infrastructure must also be taken into consideration^4^. Thus, a flexible plan based on the above details is recommended^4,6^. Therefore, we tried to established the vitrectomy timing individualization system for ocular trauma (VTISOT).

The tPVR is a one of the leading causes of blindness following ocular trauma^14,15^. Despite tremendous advancements in surgical and pharmaceutical approaches for management of tPVR, it continues to remain a clinical challenge. Moreover, there is no objective and systematic basis for clinical assessment of tPVR risk factors, and no method is available to predict the incidence of tPVR in a specific injured patient. The tPVR risk factors that can be targeted for clinical intervention may represent a breakthrough in treating tPVR and a key to the vitrectomy timing individualization, so it is important to identify it first.

In our study, 6 tPVR risk factors were identified by univariate and multivariate analysis. Among them, 5 factors, including Zone 3 Injury, Z3R Laceration, Massive VH, Retinal Disorder and Type of Injury, are immutable and objective after injury. Only vitrectomy timing can be intervened clinically. Our research also demonstrates that the incidence of tPVR is decreased by early vitrectomy, which is consistent with recent research^1,4,13^.

The reported incidence of tPVR after vitrectomy varies widely (11~66.7%)^13–16^. The following reasons account for the reported differences. First, although there is a PVR grading system, the definitions of tPVR differ from each other. For example, Cardillo *et al*. defined tPVR as any intraocular cellular proliferation associated with any degree of detachment^15^. In their report, retinal detachment was a prerequisite, so the incidence of tPVR in their report was relatively low. In most other reports, retinal detachment was not necessary for the definition of tPVR. In our research, tPVR was defined as cellular membranes within the vitreous cavity or on either retinal surfaces or obvious retinal shrinkage after trauma, which covered a relatively wide range of conditions and resulted in relatively increased tPVR incidence. Second, the timings of vitrectomy differed. Wang’s research revealed a significant difference in tPVR incidence between the early surgery group (days 2–4, 6.7%) and late surgery group (days 10–14, 66.7%)^13^. Third, the severity of injuries in each reports might be different. Thus, the definition of tPVR should be standardized in future studies.

To the best of our knowledge, a suitable method to predict the incidence of tPVR has not been reported prior to this study. Our study provides a good idea for solving this problem by a simple calculation. The predicted incidence of tPVR = exp(tPVR score)/(1 + exp(tPVR score)). The ROC curve reveals that the prediction accuracy is 78.3% (95% CI: 73.1~83.4%), and the best values of sensitivity and specificity obtained with this model are 61.9% and 81.3%, respectively.

Although the values of sensitivity and specificity of these type of predictive models may be inadequate for a direct clinical use^17^, this system provides an objective foundation for surgeons to fashion individual plans and achieve patient cooperation.

Another problem that must be discussed is that this type of formulas must be validated externally. External validation aims to address the accuracy of a model in patients from a different but plausibly related population, which may be defined as a selected study population representing the underlying disease domain^18^. Nevertheless, the tPVR score is the first predictive model of tPVR based on systematic clinical analysis. Although we have not validated this model, it provides a good idea and method for predicting tPVR. The external validation of this model can be performed by our team or other interested teams in the future.

The timing of vitrectomy for ocular trauma has remained highly controversial, with the exception of IOFB and endophthalmitis at initial presentation^1–5^. Kuhn *et al*. reports that primary comprehensive reconstruction with immediate vitrectomy at “zero hour” after injury is the best option for severe trauma with high PVR risk^1,4,19^. The advantages of this option are numerous: the risk of a subsequent development of endophthalmitis is eliminated; if an infection is present, it can be dealt with properly; all tissue pathologies that do not represent irreversible damage may be treated; the inflammatory reaction and thus the risks of PVR and secondary ciliary body destruction are reduced, etc^4^. However, in reality, this is not always possible. Various obstacles may exist. For example, the facility might not be properly prepared to offer the full armamentarium for complex vitrectomy, at least not outside “normal business hours”. Alternatively, the surgeon has no or only limited experience of vitreoretinal techniques; or the nurse is not familiar with the equipment. In addition, corneal opacity and the risk of expulsive choroidal hemorrhage may represent obstacles for immediate vitrectomy^1,4^. If comprehensive primary reconstruction is not possible, the next best option is vitrectomy performed within 100 hours or 2–4 days of the injury^1,4^. However, some others regard “days 7–14”^2,20,21^, “days 4–10”^15^ or “within 14 days”^6^ as best timing. Some even think that the 4th week is the best option in management of a gunshot perforating eye injury^3^. Hermsen’s early study showed that the best results were achieved when vitrectomy was performed between 15 and 30 days following injury^22^.

With the advancement of vitrectomy, few ophthalmologists support the options post 2 weeks. The main controversy is concentrated on immediate (zero hour), early (within 100 h or days 2–4) or late (days 7–14) at present. To date, ophthalmologists are still confused about the best timing for traumatic vitrectomy.

In this study, both univariate and multivariate analysis showed that the timing of vitrectomy was closely related to the tPVR, and the incidence of tPVR in the early surgery group (Days 2–4, 33.3%) was considerably reduced compared with other groups (Days 5–7, 61.1%; Days 8–14, 71.8%; Past 2 Weeks, 70.0%). This finding indicates that early vitrectomy may decrease the incidence of postoperative tPVR, which is consistent with the latest studies^1,4^.

The VTISOT we established integrated the tPVR risk factors, the tPVR score and the individual ocular features affecting surgical operation and efficacy, including corneal edema, suprachoroidal hemorrhage, unsutured wounds, lens injury, vitreous hemorrhage and low IOP. The evaluation results demonstrated that the patients consistent with the VTISOT principles exhibited reduced tPVR incidence and better surgical results, including reduced incidence of tPVR, postoperative RD and iatrogenic retinal injury; increased incidence of globe survival, and better postoperative BCVA and BCVA improvement.

Some researchers have recently described the potential contribution of genetic components to PVR, suggesting that it is a complex disease in which many environmental, clinical, and genetic variables may interact^10,23–25^. Rojas J, *et al*. developed three predictive models of PVR using genetic variables only. Although none of them has greater ability than that achieved by clinical predictive formulas^24^, genetic predictive variables could be added to any of the previously established clinical formulas, to improve their predictive capability and thus allow us to have a better tool in the prophylaxis of PVR^24,25^. So, the genetic predictive variables could also be added to the tPVR prediction and the VTISOT principles in future studies.

Although the VTISOT has included all clinical factors we can consider, the improvement is still possible in the future. For example, with the inclusion of more participants and genetic factors and the performance of external validation, risk factors will be more precise, and their weights in the equation will be more precise. However, currently, clinicians, especially inexperienced surgeons, can consider the VTISOT as a reference to select the timing of vitrectomy for trauma.

In conclusion, we established the vitrectomy timing individualization system for ocular trauma (VTISOT) on the basis of tPVR risk factors and the tPVR score. The patients consistent with the VTISOT principles exhibited reduced tPVR incidence and better surgical results, providing a new idea to individualize the timing of vitrectomy after trauma. Further clinical studies will be useful to evaluate and improve the VTISOT.

## Supplementary information

Data of Characteristics

## Data Availability

The datasets generated during and/or analyzed during the current study are available from the corresponding author on reasonable request.
