# Supplementary material for: The Vitrectomy Timing Individualization System for Ocular Trauma (VTISOT)
Source: Sci Rep. 2019 Aug 30;9:12612. doi: 10.1038/s41598-019-48862-2 (PMC6717195; doi:10.1038/s41598-019-48862-2)
Supplement: Supplementary file 1 — Data of Characteristics [file 41598_2019_48862_MOESM1_ESM.pdf]

The Vitrectomy Timing Individualization System for Ocular Trauma (VTISOT)

Longhui Han[1, 2#]*, Jinchen Jia[1#], Yiming Fan[1#], Luyong Yang[1#], Zhiqiang Yue[1], Wei Zhang[1], Fang Liu[1], Huanjun Kang[1], Tao Huo[1], Shaolei Han[1], Hua Shen[1], Genquan Tian[3] & Xuemin Su[3]

[1] Hebei Provincial Eye institute, Hebei Provincial Eye Hospital, Xingtai, Hebei, 054001, China.
[2] Tianjin Medical University Eye Hospital/Eye Institute, School of Optometry and Ophthalmology, Tianjin Medical University, Tianjin, 300384, China.
[3] North China University of Science and Technology, Tangshan, Hebei, 063210, China.
[#] These authors contributed equally to this work: L.H, J.J and Y.F.

* Correspondence and requests for materials should be addressed to L.H. (email: han-longhui@163.com)

Data of Characteristics

No.	In-patient No.	ADMISSION DATE TIME	Type of Injury	Cause of Injury	PVR	zone 3	z3R	mass VH	retinal disorder	Timing of Surgery (Days)	Timing of Surgery	AGE	SEX	Eye	Type of Injury	VA Pre-Op	Cornea (clear:0/ injured:1)	hyphema (Grades0-4)	VA Pre-Op	IOP	IOP	Lens	Length of laceration	retinal disorder	Consistency	RD Post-Op	Globe survival	Intra-Op Hemorrhage	Iatrogenic retinal injury	Post-Op infection	VA Post	VA Improvement	logit(P1)	logit(P2)	F2 (0,1)	PRE 1	PRE 2
1	130133388	2013-12-29 22:31:52	Penetrating	石块击伤	0	0	0	0	0	9	3	44	Male	OS	1	2	1	0	HM	Tn	2	0	13	0	1	0	1	0	0	0	5	3	-2.1461	-2.5641	0	0.10469	0.07148
2	130133452	2014-01-03 20:10:37	Penetrating	石块击伤	0	1	0	0	1	9	3	32	Male	OS	1	1	1	0	LP	Tn	2	1	13	1	0	0	1	0	0	0	4	3	2.2845	0.069	1	0.90758	0.51723
3	130133723	2014-01-05 10:33:43	Rupture	炮炸伤	1	0	0	1	0	4	1	39	Male	OS	2	0	0	4	NLP	Tn	2	0	20	0	1	0	0	0	0	0	0	0	-3.199	-0.9361	1	0.03921	0.28169
4	140133779	2014-01-14 11:23:55	Contusion	摔伤	1	1	0	0	0	9	3	24	Male	OS	3	2	0	0	HM	12.0	2	0	0	0	0	0	1	0	0	0	4	2	2.1134	0.7333	1	0.8922	0.67553
5	140133928	2014-01-02 19:15:15	Penetrating	硬物碰伤	0	1	0	0	1	4	1	3	Male	OS	1	2	1	4	HM	T-1	3	0	10	1	1	0	1	0	0	0	5	3	-1.2199	0.069	1	0.22795	0.51723
6	140133933	2014-01-02 23:22:55	Penetrating	气泵击伤	0	0	0	1	1	4	1	28	Male	OD	1	2	1	0	HM	Tn	2	1	10	0	1	0	1	0	0	0	4	2	-2.6036	-1.0614	1	0.06891	0.25704
7	140134164	2014-01-06 13:13:06	Contusion	铁棍击伤	0	0	0	1	1	3	1	44	Male	OD	3	2	0	0	HM	32.7	3	1	0	0	1	0	1	0	0	0	5	3	-1.2134	0.4167	1	0.22911	0.60268
8	140134260	2014-01-06 09:57:41	Penetrating	刀扎伤	0	0	0	0	0	14	3	60	Male	OD	1	2	1	0	HM	T+1	3	1	3	0	1	0	1	0	0	0	5	3	-2.1461	-2.5641	0	0.10469	0.07148
9	140134474	2014-01-10 19:15:14	Rupture	木棍扎伤	1	1	0	1	1	3	1	4	Male	OD	2	2	0	0	HM	T-3	1	1	20	1	1	0	1	1	1	0	4	2	1.2317	1.697	1	0.77411	0.84514
10	140134747	2014-01-15 12:30:53	Penetrating	炮炸伤	0	0	0	0	0	13	3	39	Male	OD	1	0	1	4	NLP	T-1	1	0	17	0	1	0	1	0	0	0	5	5	-2.1461	-2.5641	0	0.10469	0.07148
11	140135406	2014-01-30 18:25:35	Contusion	炮碰伤	0	1	0	0	0	4	1	42	Male	OD	3	1	1	1	LP	Tn	2	1	0	0	1	0	1	0	0	0	3	2	-1.391	0.7333	1	0.19925	0.67553
12	140135490	2014-02-03 10:44:53	Rupture	树枝击伤	0	0	0	0	0	7	2	47	Male	OS	2	1	1	1	NLP	11.0	2	1	15	0	0	0	1	0	0	0	3	2	-2.5632	-1.6251	0	0.07155	0.1645
13	140135521	2014-02-04 15:07:22	Rupture	摔伤	1	1	0	0	0	9	3	48	Female	OS	2	2	0	0	HM	T-1	1	1	23	0	0	1	1	0	0	0	4	2	1.6891	0.1943	1	0.84411	0.54841
14	140135753	2014-02-07 23:40:27	Contusion	炮崩伤	1	1	0	1	1	3	1	63	Male	OD	3	2	0	0	HM	14.3	2	1	0	1	1	0	1	0	0	0	4	2	1.6559	2.236	1	0.83969	0.90344
15	140136338	2014-02-13 14:40:53	Rupture	摔倒	1	1	0	0	0	9	3	58	Female	OD	2	1	0	4	LP	Tn	2	1	10	0	0	1	1	0	0	0	3	2	1.6891	0.1943	1	0.84411	0.54841
16	140136437	2014-02-15 20:30:19	Contusion	炮崩伤	0	1	0	0	0	4	1	44	Female	OD	3	3	0	0	CF	20.0	2	1	0	0	1	0	1	0	0	0	5	2	-1.391	0.7333	1	0.19925	0.67553
17	140137084	2014-02-25 10:52:46	Contusion	木柴击伤	1	0	0	1	1	9	3	60	Male	OD	3	2	0	1	HM	T+1	3	1	0	0	0	0	1	0	0	0	3	1	2.291	0.4167	1	0.90813	0.60268
18	140138642	2014-03-22 11:58:15	Rupture	木棍击伤	0	1	0	0	0	4	1	27	Male	OS	2	2	0	0	HM	Tn	2	0	12	0	1	0	1	0	0	0	4	2	-1.8153	0.1943	1	0.14	0.54841
19	140138847	2014-03-25 08:42:21	Contusion	高压风管击伤	1	1	0	0	0	16	4	43	Male	OS	3	1	0	0	LP	11.3	2	1	0	0	0	1	1	0	0	0	3	2	2.2544	0.7333	1	0.90503	0.67553
20	140139048	2014-03-27 09:03:38	Contusion	摔倒后碰伤	1	1	0	0	0	9	3	58	Male	OD	3	3	0	0	0.02	12.7	2	1	0	0	0	0	1	0	0	0	5	2	2.1134	0.7333	1	0.8922	0.67553
21	140139177	2014-03-29 16:20:17	Contusion	传送带击伤	1	1	0	0	0	6	2	43	Male	OS	3	1	0	0	LP	14.0	2	1	0	0	0	1	1	0	0	0	3	2	0.7303	0.7333	1	0.67488	0.67553
22	140139384	2014-04-04 09:16:47	Penetrating	不明物击伤	1	1	0	0	1	3	1	30	Male	OS	1	1	0	1	LP	Tn	2	1	8	0	1	0	1	0	0	0	3	2	-1.2199	0.069	1	0.22795	0.51723
23	140139539	2014-04-07 15:54:47	Penetrating	钻头击伤	1	1	0	0	0	4	1	22	Male	OD	1	2	0	0	HM	Tn	2	1	16	0	1	0	1	0	0	0	5	3	-2.7812	-0.7448	1	0.05835	0.32196
24	140139901	2014-04-10 12:19:59	Contusion	钢丝弹伤	0	0	0	1	1	4	1	41	Female	OD	3	4	0	1	0.12	Tn	2	0	0	0	1	0	1	0	0	0	5	1	-1.2134	0.4167	1	0.22911	0.60268
25	140139963	2014-04-13 19:32:24	Penetrating	铁片击伤	0	1	0	0	0	3	1	30	Male	OD	1	2	0	0	HM	Tn	2	1	20	0	1	0	1	0	0	0	6	4	-2.7812	-0.7448	1	0.05835	0.32196
26	140140645	2014-04-22 22:15:26	Rupture	他人手抓伤	1	1	0	0	0	10	3	66	Female	OS	2	1	0	0	LP	Tn	2	1	12	0	0	0	1	0	0	0	4	3	1.6891	0.1943	1	0.84411	0.54841
27	140142643	2014-06-03 16:50:33	Penetrating	剪刀扎伤	1	1	0	0	1	3	1	20	Female	OD	1	2	0	1	HM	T-1	1	0	14	0	1	0	1	0	0	0	6	4	-1.2199	0.069	1	0.22795	0.51723
28	140142660	2014-05-31 10:04:52	Rupture	木棍击伤	0	0	0	1	0	4	1	65	Female	OD	2	1	0	1	LP	9.0	1	1	20	0	1	0	1	0	0	0	3	2	-3.199	-0.9361	1	0.03921	0.28169
29	140142981	2014-06-08 17:01:45	Penetrating	爆炸的铁管炸伤	0	1	0	0	0	4	1	55	Male	OD	1	1	1	1	LP	T+1	3	0	14	0	1	0	1	0	0	0	3	2	-2.7812	-0.7448	1	0.05835	0.32196
30	140143102	2014-06-09 20:25:36	Penetrating	钢筋击伤	0	1	0	1	1	4	1	54	Male	OS	1	1	1	1	LP	Tn	2	1	9	2	1	0	1	1	1	0	4	3	0.2657	0.758	1	0.56603	0.68091
31	140143113	2014-06-10 17:11:29	Rupture	拳头击伤	0	1	0	0	0	3	1	42	Male	OS	2	0	0	4	NLP	T-1	1	0	15	0	1	0	1	0	0	0	3	3	-1.8153	0.1943	1	0.14	0.54841
32	140143247	2014-06-12 17:12:50	Penetrating	拳头击伤	0	1	0	0	0	4	1	21	Male	OS	1	2	1	0	HM	16.7	2	1	15	0	1	0	1	0	0	0	5	3	-2.7812	-0.7448	1	0.05835	0.32196
33	140143264	2014-06-14 00:39:44	Rupture	牛角顶伤	0	0	0	1	0	4	1	37	Male	OD	2	2	1	1	HM	T+1	3	1	20	0	1	0	1	0	0	0	4	2	-3.199	-0.9361	1	0.03921	0.28169
34	140143297	2014-06-15 13:54:49	Rupture	铁扳手打伤	0	0	0	1	0	4	1	27	Male	OS	2	2	0	0	HM	Tn	2	1	17	0	1	0	1	0	0	0	4	2	-3.199	-0.9361	1	0.03921	0.28169
35	140143457	2014-06-18 15:24:16	Rupture	硬物碰伤	0	1	0	0	0	4	1	41	Male	OS	2	1	0	0	LP	T-1	1	1	15	0	1	0	1	0	0	0	3	2	-1.8153	0.1943	1	0.14	0.54841
36	140143550	2014-06-21 19:32:59	Penetrating	铁丝弹伤	0	0	1	0	1	4	1	51	Male	OS	1	2	1	0	HM	Tn	2	1	4	0	1	0	1	0	0	0	5	3	-0.8071	0.9246	1	0.30852	0.71598
37	140144186	2014-07-03 05:10:56	Penetrating	碎玻璃打伤	0	1	0	0	0	9	3	56	Male	OD	1	2	1	0	HM	T-1	1	1	9	0	0	0	1	0	0	0	6	4	0.7231	-0.7448	1	0.67329	0.32196
38	140144485	2014-07-04 09:35:55	Rupture	砂轮片击伤	1	1	0	1	1	7	2	54	Male	OD	2	1	1	1	LP	Tn	2	0	12	1	0	1	1	0	0	0	1	0	3.353	1.697	1	0.9662	0.84514
39	140144518	2014-07-05 12:23:27	Rupture	凳子碰伤	1	1	0	1	1	4	1	51	Female	OS	2	1	1	0	LP	Tn	2	1	25	1	1	0	1	0	0	0	3	2	1.2317	1.697	1	0.77411	0.84514
40	140144526	2014-07-05 21:32:36	Perforating	铁块击伤	1	1	0	1	1	9	3	25	Male	OS	4	1	0	1	LP	T-1	1	1	10	2	0	1	0	0	0	0	0	-1	7.2893	3.0108	1	0.99932	0.95306
41	140145195	2014-07-14 15:30:34	Rupture	木块击伤	1	1	0	1	1	3	1	47	Male	OD	2	2	0	1	HM	8.5	1	0	7	2	1	0	1	0	0	0	4	2	1.2317	1.697	1	0.77411	0.84514
42	140145370	2014-07-18 16:11:09	Penetrating	铁钩击伤	1	0	1	0	1	4	2	62	Male	OS	1	1	1	1	LP	Tn	2	1	2	1	0	1	1	1	1	0	4	3	1.3143	0.9246	1	0.78823	0.71598
43	140145566	2014-07-21 15:12:50	Penetrating	玻璃碰伤	0	1	0	0	0	4	1	14	Male	OD	1	2	1	0	HM	T-1	1	1	26	0	1	0	1	0	0	0	6	4	-2.7812	-0.7448	1	0.05835	0.32196
44	140146240	2014-08-02 11:28:24	Rupture	车祸	1	1	0	1	1	4	1	51	Male	OD	2	1	1	1	LP	T-1	1	1	12	2	1	0	1	0	0	0	4	3	1.2317	1.697	1	0.77411	0.84514
45	140146910	2014-08-14 11:55:15	Contusion	铁块溅伤	1	0	0	1	0	12	3	63	Male	OS	3	4	0	0	0.08	Tn	2	1	0	0	0	0	1	0	0	0	5	1	0.7297	-0.3971	1	0.67473	0.40202
46	140147703	2014-08-25 09:20:50	Penetrating	铁片击伤	0	0	1	0	1	4	1	49	Male	OS	1	1	0	4	LP	T-1	1	1	15	0	1	0	1	0	0	0	4	3	-0.8071	0.9246	1	0.30852	0.71598
47	140147781	2014-08-27 11:18:03	Contusion	木板击伤	1	1	0	1	0	13	3	36	Male	OS	3	2	0	0	HM	11.7	2	1	0	0	0	0	1	0	1	0	4	2	3.5989	1.4223	1	0.97338	0.8057
48	140147967	2014-09-03 22:15:40	Contusion	炮崩伤	0	1	0	0	1	4	1	21	Male	OS	3	1	0	0	LP	T-1	1	1	0	1	1	0	1	0	0	0	1	0	0.1703	1.547	1	0.54248	0.82448
49	140148287	2014-09-10 18:45:52	Rupture	铁管击伤	1	1	0	0	1	4	1	45	Male	OS	2	1	1	1	LP	Tn	2	1	11	0	1	1	1	0	0	0	3	2	-0.2539	1.008	1	0.43686	0.73263
50	140148478	2014-09-13 19:57:10	Rupture	竹竿戳伤	1	0	1	1	1	5	2	55	Male	OS	2	1	1	1	LP	Tn	2	1	15	0	0	1	1	0	0	0	3	2	3.7658	2.5526	1	0.97738	0.92775
51	140148547	2014-09-15 10:46:50	Rupture	摇把碰伤	0	0	0	1	0	4	1	44	Male	OS	2	2	0	1	HM	19.3	2	1	15	0	1	0	1	0	0	0	5	3	-3.199	-0.9361	1	0.03921	0.28169
52	140148828	2014-09-21 17:36:24	Rupture	铁块击伤	0	1	0	0	1	4	1	33	Male	OD	2	1	1	0	LP	Tn	2	0	25	1	1	0	1	0	0	0	3	2	-0.2539	1.008	1	0.43686	0.73263
53	140148999	2014-09-23 22:54:46	Penetrating	改锥戳伤	1	0	1	0	1	4	3	27	Female	OD	1	1	1	0	LP	Tn	2	1	14	0	0	1	1	0	0	0	4	3	2.6973	0.9246	1	0.93687	0.71598
54	140149413	2014-09-28 21:32:07	Contusion	摔伤	0	0	0	0	0	14	3	59	Male	OD	3	3	1	0	CF	16.0	2	1	0	0	1	0	1	0	0	0	6	3	-0.7559	-1.0861	0	0.31954	0.25236
55	140149538	2014-10-04 04:59:42	Penetrating	刀砍伤	1	1	0	0	0	8	3	37	Male	OD	1	2	1	0	HM	T-1	1	1	18	0	0	0	1	0	1	0	4	2	0.7231	-0.7448	1	0.67329	0.32196
56	140149728	2014-10-14 13:16:12	Penetrating	钢丝扎伤	0	1	0	0	0	4	1	23	Male	OD	1	2	1	1	HM	Tn	2	1	3	0	1	0	1	0	0	0	4	2	-2.7812	-0.7448	1	0.05835	0.32196
57	140149852	2014-10-11 10:08:49	Penetrating	铁钩击伤	1	0	1	0	1	3	1	45	Male	OS	1	4	1	0	0.06	Tn	2	1	7	0	1	0	1	0	1	0	5	1	-0.8071	0.9246	1	0.30852	0.71598
58	140150294	2014-10-20 12:03:58	Penetrating	切割片碎片击伤	0	1	0	0	0	4	1	35	Male	OS	1	1	1	1	LP	Tn	2	0	20	0	1	0	1	1	0	0	4	3	-2.7812	-0.7448	1	0.05835	0.32196
59	140150526	2014-10-24 13:08:04	Rupture	石头砸伤	1	1	0	1	1	18	4	55	Male	OS	2	2	0	0	HM	Tn	2	1	8	1	0	1	1	0	1	0	4	2	4.8771	1.697	1	0.99244	0.84514
60	140150635	2014-10-26 17:28:51	Rupture	摇把碰伤	0	0	0	0	0	7	2	49	Male	OD	2	1	0	0	LP	Tn	2	1	20	0	0	0	1	0	0	0	2	1	-2.5632	-1.6251	0	0.07155	0.1645
61	140150653	2017-09-26 00:33:24	Contusion	油漆桶铁片击伤	1	0	0	0	1	18	4	29	Male	OS	3	2	0	0	HM	Tn	2	1	0	0	0	0	1	0	0	0	4	2	0.9465	-0.2723	1	0.72041	0.43233
62	140150857	2014-11-01 15:21:00	Rupture	骑摩托车摔伤OD	1	1	0	0	0	14	3	30	Male	OD	2	0	1	3	NLP	Tn	2	0	8	0	0	0	1	0	0	0	3	3	1.6891	0.1943	1	0.84411	0.54841
63	140150888	2014-11-02 19:22:24	Penetrating	弹簧击伤后	1	1	0	1	1	9	3	62	Male	OD	1	2	1	0	HM	Tn	2	1	8	2	0	0	1	1	0	0	5	3	3.77	0.758	1	0.97747	0.68091
64	140151204	2014-11-03 19:36:30	Contusion	砂轮击伤	0	0	0	0	1	4	1	62	Male	OD	3	3	0	1	CF	31.0	3	1	0	1	1	0	1	0	0	0	5	2	-2.6989	-0.2723	1	0.06304	0.43233
65	140151991	2014-11-16 22:59:00	Rupture	他人用石头击伤	0	1	0	0	0	6	2	52	Male	OS	2	1	1	1	LP	Tn	2	1	28	0	0	0	1	0	0	0	4	3	0.3061	0.1943	1	0.57593	0.54841
66	140152557	2014-11-26 08:06:13	Rupture	铁钩击伤	0	1	0	0	0	7	2	59	Female	OD	2	1	1	3	LP	T-2	1	1	20	0	0	0	1	0	0	0	4	3	0.3061	0.1943	1	0.57593	0.54841
67	140152621	2014-11-25 14:58:45	Penetrating	树枝扎伤	0	1	0	0	1	3	1	51	Male	OD	1	1	1	1	LP	Tn	2	0	4	1	1	0	1	0	0	0	4	3	-1.2199	0.069	1	0.22795	0.51723
68	140153280	2014-12-09 19:32:19	Penetrating	铁丝扎伤	1	0	0	0	0	12	3	46	Male	OS	1	2	1	0	HM	Tn	2	0	4	0	1	1	1	0	0	0	5	3	-2.1461	-2.5641	0	0.10469	0.07148
69	140153886	2014-12-21 13:09:13	Rupture	钢筋碰伤	0	0	0	0	0	5	2	40	Male	OD	2	2	0	0	HM	Tn	2	0	15	0	0	0	1	0	0	0	4	2	-2.5632	-1.6251	0	0.07155	0.1645
70	140154158	2014-12-24 13:17:35	Contusion	木柴碰伤	1	0	0	1	0	10	3	68	Male	OS	3	3	1	0	CF	14.0	2	1	0	0	0	0	1	0	0	0	4	1	0.7297	-0.3971	1	0.67473	0.40202
71	140154270	2014-12-28 16:30:33	Rupture	木块击伤	0	0	0	0	0	5	2	61	Male	OS	2	2	1	1	HM	T+1	3	1	15	0	0	0	1	0	0	0	4	2	-2.5632	-1.6251	0	0.07155	0.1645
72	140154282	2014-12-28 20:03:33	Penetrating	铁钉打伤	1	0	1	0	1	9	3	45	Male	OS	1	3	1	0	CF	Tn	2	1	11	0	0	1	1	0	0	0	5	2	2.6973	0.9246	1	0.93687	0.71598
73	150154940	2015-01-09 20:16:00	Contusion	石块击伤	0	0	0	0	0	13	3	51	Male	OD	3	2	1	0	HM	32.0	3	1	0	0	1	0	1	0	0	0	5	3	-0.7559	-1.0861	0	0.31954	0.25236
74	150154993	2015-01-09 17:13:09	Penetrating	铅笔刀划伤	0	1	0	0	0	6	2	11	Male	OD	1	2	0	0	HM	Tn	2	1	15	0	0	0	1	0	0	0	5	3	-0.6599	-0.7448	1	0.34076	0.32196
75	150155458	2015-01-21 13:22:03	Penetrating	刀片划伤	0	1	0	0	0	9	3	51	Male	OS	1	2	1	1	HM	T-2	1	1	10	0	0	0	1	0	0	0	5	3	0.7231	-0.7448	1	0.67329	0.32196
76	150155840	2015-01-25 19:21:15	Rupture	自行车车把碰伤	0	0	0	1	0	4	1	64	Male	OD	2	2	0	4	HM	Tn	2	1	22	0	1	0	1	0	0	0	5	3	-3.199	-0.9361	1	0.03921	0.28169
77	150156045	2015-02-11 22:09:40	Penetrating	钉子扎伤	1	1	0	0	0	12	3	34	Male	OD	1	2	1	1	HM	T-1	1	1	7	0	0	0	1	0	0	0	4	2	0.7231	-0.7448	1	0.67329	0.32196
78	150156174	2015-02-15 21:28:19	Penetrating	木棍扎伤	0	1	0	0	0	4	1	6	Male	OD	1	1	1	1	LP	T-1	1	0	17	0	1	0	1	0	0	0	4	3	-2.7812	-0.7448	1	0.05835	0.32196
79	150156297	2015-02-22 16:53:31	Contusion	炮炸伤	0	0	0	1	1	4	1	9	Male	OD	3	2	1	0	HM	T-1	1	1	0	0	1	0	1	0	0	0	4	2	-1.2134	0.4167	1	0.22911	0.60268
80	150157010	2015-01-30 08:30:40	Penetrating	铳子击伤	0	1	0	0	1	4	1	34	Male	OS	1	1	1	1	NLP	Tn	2	1	31	1	1	0	1	0	0	0	3	2	-1.2199	0.069	1	0.22795	0.51723
81	150157330	2015-02-02 17:46:32	Penetrating	钢筋打伤	1	1	0	1	1	4	1	40	Male	OS	1	1	0	1	LP	Tn	2	1	19	2	1	0	1	0	0	0	2	1	0.2657	0.758	1	0.56603	0.68091
82	150157345	2015-02-03 22:41:12	Contusion	玻璃杯碰伤	0	1	0	0	0	3	1	31	Male	OD	3	2	1	0	HM	T+1	3	1	0	0	1	0	1	1	0	0	5	3	-1.391	0.7333	1	0.19925	0.67553
83	150157380	2015-02-02 11:04:47	Contusion	扳手击伤	1	1	0	1	1	10	3	63	Male	OD	3	2	1	0	HM	T-1	1	1	0	2	0	1	0	0	0	0	0	-2	5.1603	2.236	1	0.99429	0.90344
84	150159011	2015-03-11 14:52:45	Contusion	高压水管击伤	0	1	0	0	0	7	2	25	Male	OD	3	3	0	0	CF	23.0	3	1	0	0	0	1	1	0	0	0	4	1	0.7303	0.7333	1	0.67488	0.67553
85	150159161	2015-03-12 14:23:05	Rupture	骑电动车摔伤	0	1	0	0	1	11	3	49	Female	OS	2	2	1	0	HM	Tn	2	0	15	0	0	1	0	0	0	0	0	-2	3.2505	1.008	1	0.96269	0.73263
86	150161033	2015-04-11 15:07:08	Contusion	砂轮球击碎镜片击伤OS	0	0	0	0	0	13	3	50	Male	OS	3	2	1	0	HM	T-1	1	1	0	0	1	0	1	0	0	0	5	3	-0.7559	-1.0861	0	0.31954	0.25236
87	150162307	2015-05-01 00:13:17	Contusion	摔伤	0	1	0	1	1	4	1	59	Female	OS	3	2	1	0	HM	T+2	3	1	0	1	1	0	1	0	0	0	5	3	1.6559	2.236	1	0.83969	0.90344
88	150162314	2015-05-01 19:49:56	Rupture	摔伤	0	0	0	0	0	6	2	61	Male	OS	2	1	0	1	LP	Tn	2	1	20	0	0	1	1	0	0	0	4	3	-2.5632	-1.6251	0	0.07155	0.1645
89	150163170	2015-05-12 17:44:49	Contusion	切割片打伤	1	1	0	0	0	11	3	49	Male	OS	3	2	1	1	HM	Tn	2	1	0	0	0	1	1	0	0	0	4	2	2.1134	0.7333	1	0.8922	0.67553
90	150163173	2015-05-12 18:08:24	Rupture	桌角	1	1	0	1	0	9	3	66	Male	OD	2	1	0	0	LP	Tn	2	1	10	0	0	1	1	0	0	0	3	2	3.1747	0.8833	1	0.95987	0.7075
91	150163572	2015-05-22 22:44:08	Penetrating	钢筋弹伤	0	1	0	0	1	4	1	24	Male	OD	1	1	0	3	LP	Tn	2	0	16	1	1	0	1	0	0	0	4	3	-1.2199	0.069	1	0.22795	0.51723
92	150164247	2015-05-31 15:27:51	Penetrating	石块击伤	1	1	0	0	1	5	2	32	Male	OS	1	2	1	0	HM	Tn	2	1	15	0	0	0	1	0	0	0	5	3	0.9014	0.069	1	0.71125	0.51723
93	150164552	2015-06-06 13:56:58	Rupture	摔伤	0	0	0	0	0	4	1	60	Female	OS	2	1	1	1	LP	T+1	3	1	10	0	0	0	1	0	0	0	4	3	-4.6845	-1.6251	0	0.00915	0.1645
94	150164579	2015-06-08 11:07:58	Contusion	不明物击伤	1	0	0	0	1	21	4	31	Male	OD	3	2	1	0	HM	Tn	2	1	0	0	0	0	1	0	0	0	4	2	0.9465	-0.2723	1	0.72041	0.43233
95	150165143	2015-06-22 14:36:05	Rupture	轴承击伤	0	1	0	0	1	4	1	39	Male	OS	2	3	1	0	CF	Tn	2	1	21	1	1	0	1	0	0	0	6	3	-0.2539	1.008	1	0.43686	0.73263
96	150165471	2015-06-26 10:54:37	Penetrating	铁丝扎伤	0	1	0	0	0	3	1	53	Male	OS	1	3	1	0	CF	10.3	1	1	3	0	1	0	1	0	0	0	6	3	-2.7812	-0.7448	1	0.05835	0.32196
97	150165492	2015-06-26 21:00:52	Contusion	钉子弹伤	0	0	0	0	0	14	3	44	Male	OD	3	3	1	0	CF	23.7	3	1	0	0	1	0	1	0	0	0	6	3	-0.7559	-1.0861	0	0.31954	0.25236
98	150165903	2015-07-03 10:32:25	Rupture	轴承击伤	1	1	0	0	0	7	2	24	Male	OS	2	1	1	0	LP	Tn	2	1	14	0	0	0	1	0	0	0	3	2	0.3061	0.1943	1	0.57593	0.54841
99	150166084	2015-07-06 16:33:03	Contusion	高压水击伤	1	0	0	0	1	9	3	27	Male	OS	3	3	1	0	CF	14.0	2	1	0	0	0	0	1	0	0	0	5	2	0.8054	-0.2723	1	0.69114	0.43233
100	150166843	2015-07-16 03:36:02	Rupture	钢筋碰伤	0	1	0	0	0	4	1	56	Male	OS	2	2	0	1	HM	Tn	2	0	9	0	1	0	1	0	0	0	4	2	-1.8153	0.1943	1	0.14	0.54841
101	150167332	2015-07-22 16:08:26	Contusion	断裂的高压气管击伤	1	0	0	1	1	11	3	40	Male	OS	3	3	0	0	CF	37.3	3	1	0	0	0	1	1	0	0	0	4	1	2.291	0.4167	1	0.90813	0.60268
102	150167370	2015-07-23 11:50:58	Rupture	铁棍碰伤	1	1	0	0	0	4	3	65	Male	OD	2	2	1	1	HM	Tn	2	1	11	0	0	0	1	0	0	0	4	2	1.6891	0.1943	1	0.84411	0.54841
103	150167536	2015-07-29 20:38:02	Rupture	车马槽碰伤	1	1	0	0	0	9	3	40	Male	OS	2	2	1	1	HM	14.0	2	0	30	0	0	1	1	0	1	0	4	2	1.6891	0.1943	1	0.84411	0.54841
104	150167712	2015-07-28 15:15:43	Penetrating	钢筋划伤	1	1	0	1	1	10	3	37	Female	OD	1	0	1	1	NLP	Tn	2	0	15	0	0	0	1	0	1	0	4	4	2.2845	0.069	1	0.90758	0.51723
105	150168982	2015-08-18 21:15:18	Penetrating	剪刀扎伤	1	1	0	0	1	3	1	27	Female	OD	1	3	1	0	CF	Tn	2	1	16	0	1	0	1	0	0	0	4	1	-1.2199	0.069	1	0.22795	0.51723
106	150169231	2015-08-23 15:48:49	Penetrating	钉子击伤	1	0	1	1	1	9	1	33	Male	OS	1	2	1	0	HM	T+1	3	1	11	0	1	0	1	1	0	0	6	4	0.6785	1.6136	1	0.66341	0.83391
107	150169525	2015-08-28 13:00:40	Penetrating	铁丝扎伤	1	0	1	0	1	3	3	25	Male	OS	1	2	1	0	HM	Tn	2	1	4	0	0	0	1	0	0	0	3	1	2.6973	0.9246	1	0.93687	0.71598
108	150169527	2015-08-28 19:29:38	Penetrating	树枝扎伤	1	1	0	0	0	4	3	64	Male	OS	1	1	1	4	LP	T+1	3	1	5	0	0	0	1	0	0	0	4	3	0.7231	-0.7448	1	0.67329	0.32196
109	150169705	2015-08-29 16:36:57	Rupture	他人拳头击伤	1	1	0	1	1	4	1	30	Male	OS	2	1	1	0	LP	Tn	2	1	20	1	1	1	1	0	0	0	3	2	1.2317	1.697	1	0.77411	0.84514
110	150169709	2015-08-29 19:49:11	Rupture	他人拳击伤	1	1	0	1	1	5	2	60	Male	OS	2	1	1	1	LP	T+1	3	1	20	1	0	0	1	0	0	0	3	2	3.353	1.697	1	0.9662	0.84514
111	150170727	2015-09-19 20:45:01	Contusion	轮胎爆炸伤	0	1	0	0	0	4	1	39	Male	OD	3	1	1	1	LP	26.0	3	1	0	0	1	0	1	0	0	0	3	2	-1.391	0.7333	1	0.19925	0.67553
112	150170958	2015-09-21 17:30:19	Penetrating	角铁碰伤	0	1	0	0	0	4	1	26	Female	OS	1	0	1	3	NLP	T-1	1	1	17	0	1	0	1	0	1	0	4	4	-2.7812	-0.7448	1	0.05835	0.32196
113	150170966	2015-09-22 06:22:53	Rupture	锚杆机手柄打伤	1	1	0	1	1	4	1	45	Male	OD	2	1	0	0	LP	T-1	1	1	15	3	1	0	1	0	0	0	2	1	1.2317	1.697	1	0.77411	0.84514
114	150171133	2015-10-04 12:54:05	Rupture	玉米击伤	1	1	0	0	0	11	3	37	Female	OS	2	2	0	1	HM	Tn	2	1	20	0	0	0	1	0	0	0	4	2	1.6891	0.1943	1	0.84411	0.54841

115	150171294	2015-10-02 14:22:04	Penetrating	眼镜片碎玻璃扎伤	1	1	0	0	0	12	3	29	Male	OD	1	2	1	1	HM	T-1	1	1	6	0	0	0	1	1	0	0	5	3	0.7231	-0.7448	1	0.67329	0.32196
116	150171498	2015-10-08 15:40:14	Contusion	玉米击伤	1	1	0	1	1	4	1	49	Female	OS	3	2	0	0	HM	Tn	2	1	0	0	1	0	1	0	0	0	5	3	1.6559	2.236	1	0.83969	0.90344
117	150172262	2015-10-20 11:55:35	Rupture	桌子角	0	1	0	0	0	4	1	7	Male	OD	2	1	1	0	LP	Tn	2	1	14	0	1	0	1	0	0	0	4	3	-1.8153	0.1943	1	0.14	0.54841
118	150172351	2015-10-25 18:31:47	Penetrating	钉子扎伤	0	1	0	0	0	3	1	47	Male	OD	1	2	1	0	HM	Tn	2	1	3	0	1	0	1	0	0	0	4	2	-2.7812	-0.7448	1	0.05835	0.32196
119	150172463	2015-10-20 15:04:44	Penetrating	钉子扎伤	1	0	1	1	1	6	2	34	Male	OD	1	2	1	0	HM	Tn	2	1	7	0	0	0	1	0	0	0	4	2	2.7998	1.6136	1	0.94267	0.83391
120	150172614	2015-10-23 15:50:26	Perforating	铁屑	0	1	0	0	0	3	1	38	Male	OS	4	3	0	0	CF	Tn	2	0	6	0	1	0	1	1	0	0	5	2	0.738	1.5081	1	0.67657	0.81878
121	150172899	2015-10-30 10:37:48	Contusion	砂轮切割片击伤	1	1	0	0	0	5	2	53	Male	OD	3	5	0	0	0.25	Tn	2	1	0	0	0	0	1	1	0	0	6	1	0.7303	0.7333	1	0.67488	0.67553
122	150173057	2015-11-01 10:10:07	Penetrating	钉子击伤	1	0	1	0	1	11	3	52	Male	OS	1	2	0	0	HM	Tn	2	1	10	0	0	1	1	0	0	0	5	3	2.6973	0.9246	1	0.93687	0.71598
123	150173259	2015-11-03 14:39:18	Contusion	骑电动车碰伤	0	1	0	0	1	4	1	57	Male	OS	3	1	0	0	LP	19.0	2	1	0	1	1	0	1	0	0	0	3	2	0.1703	1.547	1	0.54248	0.82448
124	150173357	2015-11-05 08:15:00	Penetrating	铁屑	0	1	0	0	0	4	1	31	Male	OS	1	4	0	0	0.04	Tn	2	1	4	0	1	0	1	0	0	0	5	1	-2.7812	-0.7448	1	0.05835	0.32196
125	150173390	2015-11-05 14:55:28	Penetrating	木块击伤	0	1	0	0	0	7	2	60	Male	OD	1	1	0	0	LP	T-1	1	1	13	0	0	0	1	0	0	0	4	3	-0.6599	-0.7448	1	0.34076	0.32196
126	150173767	2015-11-13 10:23:07	Contusion	碰伤	0	1	0	0	0	4	1	33	Female	OS	3	1	1	4	LP	T-1	1	1	0	0	1	0	1	0	0	0	3	2	-1.391	0.7333	1	0.19925	0.67553
127	150174124	2015-11-21 17:11:04	Contusion	摔伤	1	1	0	0	0	9	3	61	Male	OD	3	3	0	0	0.02	Tn	2	1	0	0	0	1	1	0	0	0	5	2	2.1134	0.7333	1	0.8922	0.67553
128	150175575	2015-12-16 09:50:34	Rupture	磕于水壶把	1	1	0	1	1	10	3	41	Male	OS	2	2	0	0	HM	Tn	2	1	24	1	0	0	0	0	0	0	0	-2	4.736	1.697	1	0.9913	0.84514
129	150175659	2015-12-20 10:16:22	Rupture	木块击伤	1	1	0	0	0	7	2	26	Male	OS	2	1	1	4	LP	T-2	1	0	15	0	0	0	1	0	1	0	1	0	0.3061	0.1943	1	0.57593	0.54841
130	150175674	2015-12-20 19:35:28	Penetrating	切割片击伤	0	1	0	0	0	4	1	59	Male	OS	1	1	1	1	LP	T-1	1	1	25	0	1	0	1	0	0	0	4	3	-2.7812	-0.7448	1	0.05835	0.32196
131	150175844	2016-01-03 20:20:23	Rupture	铁棍击伤	1	1	0	1	1	4	1	33	Male	OD	2	1	1	3	LP	T-2	1	1	25	2	1	1	1	0	0	0	3	2	1.2317	1.697	1	0.77411	0.84514
132	150175844	2016-01-03 20:20:23	Rupture	铁棍击伤	1	1	0	1	1	13	3	33	Male	OS	2	1	1	3	LP	T-1	1	1	37	1	0	0	0	0	0	0	0	-1	4.736	1.697	1	0.9913	0.84514
133	150176111	2015-12-24 19:43:38	Rupture	铁丝及机器崩伤	1	1	0	1	1	9	3	27	Male	OS	2	2	0	0	HM	Tn	2	0	28	1	0	1	1	0	0	0	3	1	4.736	1.697	1	0.9913	0.84514
134	150176145	2015-12-26 15:54:27	Contusion	木柴击伤	0	1	0	0	0	4	1	59	Male	OD	3	3	0	0	CF	24.5	3	1	0	0	1	0	1	0	0	0	5	2	-1.391	0.7333	1	0.19925	0.67553
135	160175845	2016-01-04 12:11:35	Penetrating	铁片击伤	1	1	0	0	0	14	3	44	Male	OD	1	3	1	0	CF	Tn	2	1	19	0	0	0	1	0	0	0	5	2	0.7231	-0.7448	1	0.67329	0.32196
136	160176025	2016-01-02 15:38:25	Contusion	砂轮击伤	1	0	0	1	1	12	3	26	Male	OD	3	1	1	1	LP	32.0	3	1	0	0	0	0	1	0	0	0	3	2	2.291	0.4167	1	0.90813	0.60268
137	160177077	2016-01-16 15:39:27	Contusion	他人拳头打伤	1	0	0	1	0	6	2	68	Male	OS	3	1	1	0	LP	13.0	2	1	0	0	0	0	1	0	0	0	3	2	-0.6534	-0.3971	1	0.34223	0.40202
138	160177114	2016-01-11 17:05:27	Contusion	弹起木柴击伤	1	1	0	1	1	4	1	53	Male	OS	3	3	0	0	CF	Tn	2	1	0	1	1	0	1	0	0	0	4	1	1.6559	2.236	1	0.83969	0.90344
139	160178349	2016-02-03 13:33:54	Contusion	弹簧弹伤	1	1	0	1	1	3	1	57	Male	OD	3	2	0	0	HM	13.0	2	1	0	2	1	0	1	1	0	0	5	3	1.6559	2.236	1	0.83969	0.90344
140	160178463	2016-02-09 14:38:13	Contusion	炮炸伤	1	1	0	0	1	10	3	22	Female	OD	3	2	1	1	HM	T+1	3	1	0	0	0	1	1	0	0	0	4	2	3.6747	1.547	1	0.97527	0.82448
141	160178699	2016-02-13 14:53:34	Contusion	碰伤	1	0	0	0	1	13	3	56	Male	OD	3	2	1	1	HM	17.0	2	1	0	0	0	0	1	0	0	0	4	2	0.8054	-0.2723	1	0.69114	0.43233
142	160179336	2016-02-17 08:56:36	Rupture	炮炸伤	1	1	0	1	1	4	1	51	Male	OS	2	0	1	4	NLP	T-2	1	1	15	2	1	1	1	0	0	0	2	2	1.2317	1.697	1	0.77411	0.84514
143	160180166	2016-02-27 16:53:44	Penetrating	木棍扎伤	0	1	0	0	1	6	2	20	Male	OS	1	2	1	0	HM	Tn	2	1	15	1	0	0	1	0	0	0	5	3	0.9014	0.069	1	0.71125	0.51723
144	160180885	2016-03-11 22:10:19	Rupture	三轮车铁板碰伤	0	0	0	1	0	4	1	59	Male	OD	2	2	1	4	HM	43.0	3	1	8	0	1	0	1	0	0	0	4	2	-3.199	-0.9361	1	0.03921	0.28169
145	160181363	2016-03-25 21:43:54	Rupture	他人拳击伤	0	1	0	0	0	4	1	44	Male	OS	2	2	0	0	HM	Tn	2	1	8	0	1	0	1	0	0	0	5	3	-1.8153	0.1943	1	0.14	0.54841
146	160181446	2016-03-16 15:31:40	Rupture	小推车撞伤	1	0	1	1	1	6	2	44	Male	OD	2	2	1	0	HM	Tn	2	1	20	1	0	0	1	0	0	0	3	1	3.7658	2.5526	1	0.97738	0.92775
147	160182137	2016-03-26 11:42:01	Perforating	气钉击伤	1	1	0	0	0	3	1	44	Male	OS	4	5	0	0	0.40	Tn	2	0	8	0	1	1	1	0	0	0	6	1	0.738	1.5081	1	0.67657	0.81878
148	160182487	2016-03-30 12:51:54	Contusion	三角带弹伤	1	1	0	0	0	11	3	44	Male	OS	3	2	1	0	HM	37.0	3	1	0	0	0	1	1	0	1	0	4	2	2.1134	0.7333	1	0.8922	0.67553
149	160182575	2016-04-01 15:49:57	Penetrating	钢丝扎伤	1	0	1	1	1	9	1	36	Male	OS	1	2	1	0	HM	Tn	2	1	3	0	1	0	1	0	0	0	6	4	0.6785	1.6136	1	0.66341	0.83391
150	160182631	2016-04-04 21:32:30	Contusion	高压液体喷伤	1	1	0	0	0	9	3	26	Male	OD	3	2	1	0	HM	20.0	2	1	0	0	0	1	1	0	0	0	4	2	2.1134	0.7333	1	0.8922	0.67553
151	160182650	2016-04-05 08:59:37	Penetrating	玻璃划伤	1	1	0	0	0	7	2	28	Male	OS	1	2	1	0	HM	Tn	2	1	15	0	0	0	1	0	0	0	4	2	-0.6599	-0.7448	1	0.34076	0.32196
152	160183451	2016-04-15 23:19:05	Penetrating	钢筋击伤	0	1	0	0	1	2	1	55	Male	OS	1	1	1	0	LP	T-1	1	1	16	0	1	0	1	0	0	0	2	1	-1.2199	0.069	1	0.22795	0.51723
153	160184040	2016-04-23 14:40:42	Contusion	碰伤	1	1	0	0	0	4	1	62	Female	OS	3	3	1	0	0.01	19.0	2	1	0	0	1	1	1	0	0	0	4	1	-1.391	0.7333	1	0.19925	0.67553
154	160184131	2016-04-24 19:11:34	Penetrating	刀划伤	0	0	0	0	0	11	3	5	Male	OD	1	1	1	0	LP	Tn	2	1	19	0	1	0	1	0	0	0	5	4	-2.1461	-2.5641	0	0.10469	0.07148
155	160184296	2016-04-26 14:12:10	Rupture	木棍击伤	0	0	0	1	1	7	2	66	Female	OD	2	2	0	2	HM	25.0	3	1	18	1	0	0	1	0	0	0	3	1	0.4837	-0.1224	1	0.61863	0.46945
156	160184372	2016-04-29 13:33:43	Penetrating	他人用木棍扎伤	1	1	0	0	0	7	2	10	Female	OS	1	1	1	0	LP	Tn	2	1	11	0	0	0	1	0	0	0	4	3	-0.6599	-0.7448	1	0.34076	0.32196
157	160184380	2016-04-30 09:55:43	Rupture	车门把手击伤	1	1	0	1	1	5	2	37	Male	OS	2	0	1	3	NLP	T-1	1	1	15	0	0	0	1	0	0	0	2	2	1.8674	1.008	1	0.86616	0.73263
158	160184497	2016-04-30 21:17:37	Penetrating	玻璃	1	0	1	0	1	9	1	44	Male	OS	1	2	1	0	HM	Tn	2	0	2	0	1	1	1	0	0	0	6	4	-0.8071	0.9246	1	0.30852	0.71598
159	160184521	2016-05-08 07:15:08	Penetrating	钢丝扎伤	1	0	1	0	1	8	3	45	Male	OS	1	2	1	0	HM	Tn	2	0	3	0	0	1	1	0	0	0	6	4	2.6973	0.9246	1	0.93687	0.71598
160	160184547	2016-05-01 15:52:57	Penetrating	钢丝弹伤	1	0	1	0	1	11	3	47	Male	OD	1	2	1	0	HM	22.0	3	1	4	0	0	0	1	0	0	0	6	4	2.6973	0.9246	1	0.93687	0.71598
161	160184763	2016-05-06 14:45:22	Contusion	木块击伤	0	1	0	1	1	4	1	68	Male	OD	3	2	1	0	HM	Tn	2	1	0	1	1	0	1	1	0	0	4	2	1.6559	2.236	1	0.83969	0.90344
162	160185005	2016-05-07 11:20:30	Contusion	铁钉碰伤	1	1	0	1	1	4	1	65	Male	OD	3	3	0	0	CF	25.0	3	1	0	1	1	0	1	0	0	0	4	1	1.6559	2.236	1	0.83969	0.90344
163	160185162	2016-05-09 18:16:44	Rupture	摔倒后碰伤	1	0	0	0	0	4	1	48	Male	OS	2	3	1	0	CF	34.6	3	1	13	0	0	1	1	0	0	0	5	2	-4.6845	-1.6251	0	0.00915	0.1645
164	160186275	2016-05-29 00:18:58	Rupture	啤酒瓶砸伤	0	1	0	0	0	4	1	51	Female	OS	2	4	0	0	0.04	Tn	2	1	10	0	1	0	1	0	0	0	5	1	-1.8153	0.1943	1	0.14	0.54841
165	160186586	2016-06-01 10:20:34	Rupture	钢丝弹伤	1	1	0	1	1	10	3	42	Male	OD	2	3	1	0	CF	Tn	2	1	6	1	0	0	1	0	0	0	5	2	4.736	1.697	1	0.9913	0.84514
166	160186842	2016-06-07 16:39:35	Contusion	木柴击伤	1	1	0	0	1	6	2	69	Male	OD	3	4	1	0	0.10	48.0	3	1	0	0	0	0	1	0	0	0	5	1	2.2917	1.547	1	0.90819	0.82448
167	160187049	2016-06-13 16:09:48	Contusion	高压瓶气绳击伤	1	1	0	0	0	9	3	46	Male	OD	3	2	0	1	HM	12.5	2	1	0	0	0	1	1	0	0	0	4	2	2.1134	0.7333	1	0.8922	0.67553
168	160187057	2015-02-02 11:04:47	Contusion	扳手击伤	1	1	0	1	1	3	1	63	Male	OD	3	2	1	0	HM	T-1	1	1	0	3	1	1	1	1	1	0	4	2	1.6559	2.236	1	0.83969	0.90344
169	160187082	2016-06-15 12:59:22	Penetrating	木头击伤	1	1	0	1	1	9	3	51	Male	OS	1	2	1	1	HM	Tn	2	1	12	1	0	0	1	0	0	0	4	2	3.77	0.758	1	0.97747	0.68091
170	160187132	2016-06-14 11:22:46	Contusion	砂轮击伤	1	1	0	0	1	4	1	55	Male	OD	3	2	1	1	HM	44.0	3	1	0	1	1	0	1	0	0	0	3	1	0.1703	1.547	1	0.54248	0.82448
171	160187140	2016-06-14 15:26:24	Contusion	钢筋击伤	0	0	0	1	1	5	2	50	Male	OS	3	3	0	0	CF	21.0	2	1	0	0	0	0	1	0	0	0	4	1	0.908	0.4167	1	0.71259	0.60268
172	160187396	2016-06-20 12:35:40	Penetrating	钢筋	0	0	0	0	0	15	4	40	Male	OS	1	2	1	1	HM	Tn	2	1	18	0	0	0	1	0	0	0	4	2	-2.0051	-2.5641	0	0.11867	0.07148
173	160187716	2016-06-27 17:18:54	Rupture	手机砸伤	0	1	0	0	0	17	4	36	Female	OS	2	3	0	0	CF	Tn	2	1	11	0	0	0	1	0	0	0	3	0	1.8301	0.1943	1	0.86178	0.54841
174	160187800	2016-06-27 08:33:26	Penetrating	剪刀扎伤	0	0	1	1	1	4	1	2	Male	OD	1	2	1	0	HM	Tn	2	1	4	0	1	0	1	1	0	0	5	3	0.6785	1.6136	1	0.66341	0.83391
175	160187899	2016-06-24 09:30:25	Contusion	爆炸的塑料瓶击伤	1	1	0	1	1	14	3	39	Female	OS	3	3	0	0	CF	9.0	1	1	0	2	0	1	0	0	0	0	0	-3	5.1603	2.236	1	0.99429	0.90344
176	160188444	2016-07-06 20:13:46	Rupture	锄头把击伤	1	1	0	0	1	3	1	54	Male	OD	2	1	1	1	LP	11.7	2	1	25	1	1	1	1	0	0	0	3	2	-0.2539	1.008	1	0.43686	0.73263
177	160188539	2016-07-06 01:36:01	Rupture	凳子碰伤	0	0	0	0	0	14	3	66	Male	OD	2	1	1	0	LP	T+2	3	1	20	0	1	1	1	0	0	0	6	5	-1.1802	-1.6251	0	0.23503	0.1645
178	160188555	2016-07-05 14:43:14	Penetrating	钢钉弹伤	1	1	0	0	0	9	3	31	Male	OD	1	1	0	1	LP	Tn	2	1	6	0	0	0	1	1	0	0	4	3	0.7231	-0.7448	1	0.67329	0.32196
179	160188864	2016-07-04 19:58:45	Penetrating	啤酒瓶炸伤	0	1	0	0	0	4	1	34	Female	OD	1	2	0	0	HM	Tn	2	1	20	0	1	0	1	0	0	0	4	2	-2.7812	-0.7448	1	0.05835	0.32196
180	160189313	2016-08-03 19:12:25	Penetrating	锯片弹伤	0	1	0	0	1	4	1	51	Male	OS	1	1	1	4	LP	T-1	1	1	21	1	1	0	1	0	0	0	3	2	-1.2199	0.069	1	0.22795	0.51723
181	160189434	2016-08-02 14:53:28	Rupture	砂轮片击伤	1	1	0	0	1	5	2	33	Male	OS	2	1	1	1	LP	13.0	2	1	15	0	0	0	1	0	0	0	1	0	1.8674	1.008	1	0.86616	0.73263
182	160189664	2016-08-06 15:40:58	Rupture	烟花爆炸	1	1	0	0	0	5	2	43	Male	OS	2	1	1	4	LP	9.0	1	0	21	0	0	0	1	1	0	0	3	2	0.3061	0.1943	1	0.57593	0.54841
183	160192003	2016-08-27 08:17:55	Penetrating	钢钉弹伤	0	1	0	0	0	7	2	47	Male	OS	1	2	1	4	HM	Tn	2	1	8	0	0	0	1	0	0	0	4	2	-0.6599	-0.7448	1	0.34076	0.32196
184	160192177	2016-08-31 12:44:21	Contusion	他人拳头打伤	0	0	0	1	1	5	2	53	Male	OS	3	3	0	0	CF	Tn	2	1	0	0	0	0	1	0	0	0	5	2	0.908	0.4167	1	0.71259	0.60268
185	160192359	2016-09-02 15:28:34	Rupture	铁块碰伤	1	1	0	0	0	9	3	46	Male	OD	2	1	1	0	LP	T-1	1	1	15	0	0	0	1	0	0	0	3	2	1.6891	0.1943	1	0.84411	0.54841
186	160192668	2016-09-07 20:11:57	Rupture	摔伤	0	1	0	0	0	4	1	60	Male	OS	2	1	1	1	LP	Tn	2	1	20	0	1	0	1	0	0	0	3	2	-1.8153	0.1943	1	0.14	0.54841
187	160192837	2016-09-13 19:35:45	Contusion	木柴击伤	1	0	0	1	1	4	1	65	Male	OS	3	3	0	0	CF	17.0	2	1	0	0	1	0	1	0	0	0	4	1	-1.2134	0.4167	1	0.22911	0.60268
188	160192937	2016-09-15 09:55:12	Contusion	抛光片击伤	1	0	0	0	1	10	3	52	Male	OS	3	2	1	1	HM	14.0	2	1	0	0	0	0	1	0	0	0	5	3	0.8054	-0.2723	1	0.69114	0.43233
189	160193740	2016-09-29 17:56:40	Rupture	炮崩伤	1	1	0	1	1	9	3	31	Male	OS	2	1	1	1	LP	Tn	2	1	8	2	0	1	0	0	0	0	0	-1	4.736	1.697	1	0.9913	0.84514
190	160193742	2016-09-29 18:44:07	Penetrating	玻璃镜子扎伤	0	1	0	0	0	6	2	11	Male	OD	1	0	1	3	NLP	T-1	1	1	8	0	0	0	1	0	1	0	4	4	-0.6599	-0.7448	1	0.34076	0.32196
191	160194151	2016-10-11 19:09:23	Rupture	他人用不明物击伤	1	1	0	0	0	6	2	46	Male	OD	2	1	0	1	LP	Tn	2	0	10	0	0	0	1	0	0	0	3	2	0.3061	0.1943	1	0.57593	0.54841
192	160194764	2016-10-17 03:29:03	Rupture	旋耕犁碰伤	1	0	0	0	0	4	1	51	Male	OD	2	2	1	0	HM	T+1	3	1	18	0	0	1	1	1	1	0	4	2	-4.6845	-1.6251	0	0.00915	0.1645
193	160194873	2016-10-17 16:22:37	Contusion	弹起的钉子击伤	0	0	0	0	0	12	3	64	Male	OS	3	3	1	1	CF	11.0	2	1	0	0	1	0	1	0	0	0	5	2	-0.7559	-1.0861	0	0.31954	0.25236
194	160195008	2016-10-18 13:24:19	Penetrating	大铁钉击伤	0	1	0	0	1	3	1	44	Male	OD	1	1	1	4	LP	Tn	2	1	6	0	1	0	1	0	0	0	4	3	-1.2199	0.069	1	0.22795	0.51723
195	160195010	2016-10-18 19:16:42	Rupture	砂轮击伤	1	1	0	1	1	9	3	57	Male	OS	2	0	1	1	NLP	T-1	1	1	30	2	0	1	0	0	0	0	0	0	4.736	1.697	1	0.9913	0.84514
196	160195229	2016-10-22 23:35:05	Contusion	用钝器进行加害	0	0	0	0	0	22	4	18	Male	OD	3	3	0	0	CF	25.0	3	1	0	0	0	0	1	0	0	0	4	1	-0.6149	-1.0861	0	0.35095	0.25236
197	160195397	2016-10-26 19:39:15	Rupture	摔伤	1	1	0	1	1	3	1	47	Male	OS	2	2	1	1	HM	Tn	2	1	16	3	1	0	1	1	0	0	5	3	1.2317	1.697	1	0.77411	0.84514
198	160195702	2016-10-31 09:18:56	Rupture	铁棍击伤	0	0	0	0	0	10	3	41	Male	OS	2	2	1	0	HM	Tn	2	1	15	0	1	0	1	0	0	0	4	2	-1.1802	-1.6251	0	0.23503	0.1645
199	160195794	2016-11-04 14:33:20	Penetrating	铁丝弹伤	0	0	0	0	0	10	3	64	Male	OS	1	2	1	0	HM	Tn	2	0	3	0	1	0	1	0	0	0	6	4	-2.1461	-2.5641	0	0.10469	0.07148
200	160196220	2016-11-10 13:41:41	Rupture	木板击伤	1	1	0	1	1	7	2	60	Male	OD	2	1	1	1	LP	Tn	2	1	20	1	0	1	0	0	0	0	0	-1	3.353	1.697	1	0.9662	0.84514
201	160196413	2016-11-12 11:22:19	Penetrating	玩具枪扎伤	0	1	0	0	0	5	2	3	Male	OS	1	2	1	2	HM	Tn	2	1	11	0	0	0	1	0	0	0	5	3	-0.6599	-0.7448	1	0.34076	0.32196
202	160196446	2016-11-13 23:50:24	Rupture	木板击伤	1	0	1	1	1	11	3	57	Male	OD	2	3	0	2	CF	Tn	2	1	15	1	0	1	0	0	0	0	0	-3	5.1489	2.5526	1	0.99423	0.92775
203	160196685	2016-11-15 14:14:17	Contusion	碰伤	0	0	0	0	0	13	3	27	Male	OS	3	4	0	0	0.10	13.0	2	1	0	0	1	0	1	0	0	0	5	1	-0.7559	-1.0861	0	0.31954	0.25236
204	160197210	2016-11-25 12:24:35	Penetrating	钉子扎伤	1	0	1	0	0	9	3	33	Male	OD	1	3	0	0	CF	17.0	2	0	10	0	0	0	1	0	0	0	5	2	1.136	0.1109	1	0.75694	0.52769
205	160197492	2016-11-28 11:51:47	Contusion	木屑击伤	1	1	0	0	0	9	3	71	Female	OD	3	4	0	0	0.20	14.0	2	1	0	0	0	1	1	0	0	0	5	1	2.1134	0.7333	1	0.8922	0.67553
206	160197743	2016-12-04 12:03:03	Perforating	铁棍扎伤	1	1	0	0	0	9	1	25	Male	OS	4	3	1	0	CF	Tn	2	1	8	0	1	0	1	0	0	0	5	2	0.738	1.5081	1	0.67657	0.81878
207	160198092	2016-12-07 17:10:28	Contusion	树枝击伤	0	0	0	0	0	10	3	62	Female	OD	3	3	1	1	CF	15.0	2	1	0	0	1	0	1	0	0	0	5	2	-0.7559	-1.0861	0	0.31954	0.25236
208	160198412	2016-12-13 14:33:27	Rupture	铁板碰伤	1	1	0	1	1	5	2	60	Male	OD	2	1	0	0	LP	Tn	2	1	20	2	0	1	1	1	0	0	3	2	3.353	1.697	1	0.9662	0.84514
209	160198862	2016-12-19 17:01:04	Contusion	木头戳伤	0	1	0	0	1	3	1	70	Male	OD	3	2	0	0	HM	20.0	2	1	0	1	1	0	1	1	0	0	4	2	0.1703	1.547	1	0.54248	0.82448
210	160199179	2016-12-27 11:14:16	Rupture	铁块砸伤	0	1	0	0	0	7	2	41	Male	OS	2	3	0	0	CF	Tn	2	1	30	0	0	0	1	1	0	0	5	2	0.3061	0.1943	1	0.57593	0.54841
211	160199262	2016-12-29 17:24:19	Contusion	抛光机片崩伤	1	1	0	0	1	11	3	49	Male	OD	3	3	1	0	CF	Tn	2	1	0	0	0	1	1	0	0	0	4	1	3.6747	1.547	1	0.97527	0.82448
212	160199311	2016-12-30 14:34:51	Rupture	骑电动车碰伤OD	1	1	0	0	0	9	3	48	Male	OD	2	2	0	2	HM	Tn	2	1	45	0	0	1	1	1	0	0	4	2	1.6891	0.1943	1	0.84411	0.54841
213	160199341	2017-01-02 08:57:20	Contusion	树枝击伤	1	0	0	0	1	12	3	34	Male	OS	3	3	0	0	CM	19.0	2	1	0	0	0	1	1	0	0	0	5	2	0.8054	-0.2723	1	0.69114	0.43233
214	170199716	2017-01-07 17:20:28	Contusion	爆炸的电瓶击伤	1	1	0	0	1	9	3	24	Male	OD	3	2	1	0	HM	T-1	1	1	0	0	0	0	1	0	0	1	4	2	3.6747	1.547	1	0.97527	0.82448
215	170199746	2017-01-08 23:46:19	Contusion	烟花炸伤	1	1	0	1	1	4	1	35	Male	OD	3	1	0	0	LP	5.0	1	1	0	1	1	1	0	0	0	0	0	-1	1.6559	2.236	1	0.83969	0.90344
216	170199853	2017-01-10 10:59:40	Contusion	织布梭击伤	0	0	0	1	1	3	1	56	Male	OD	3	2	0	0	HM	Tn	2	1	0	0	1	0	1	0	0	0	5	3	-1.2134	0.4167	1	0.22911	0.60268
217	170200159	2017-01-13 14:44:02	Contusion	铁钉击伤	0	1	0	0	0	3	1	60	Male	OD	3	3	0	0	CM	20.0	2	1	0	0	1	0	1	0	0	0	5	2	-1.391	0.7333	1	0.19925	0.67553
218	170200431	2017-01-16 15:32:03	Rupture	树枝弹伤	1	1	0	1	1	9	3	48	Male	OD	2	0	0	3	NLP	Tn	2	1	15	2	0	1	1	0	0	0	2	2	4.736	1.697	1	0.9913	0.84514
219	170200524	2017-01-17 15:25:48	Perforating	石子击伤	1	1	0	1	1	11	3	29	Male	OD	4	1	0	0	LP	Tn	2	1	15	1	0	1	1	0	0	0	4	3	7.2893	3.0108	1	0.99932	0.95306
220	170200672	2017-01-20 21:54:33	Penetrating	木棍扎伤	0	1	0	0	1	4	1	44	Male	OS	1	1	1	1	LP	Tn	2	1	6	1	1	0	1	0	0	0	4	3	-1.2199	0.069	1	0.22795	0.51723
221	170200693	2017-01-21 23:39:25	Penetrating	茶杯投伤	0	1	0	0	0	10	3	28	Male	OS	1	1	1	1	LP	Tn	2	1	17	0	0	0	1	0	0	0	4	3	0.7231	-0.7448	1	0.67329	0.32196
222	170200788	2017-01-25 17:29:40	Penetrating	水果刀划伤	1	1	0	0	0	9	3	64	Male	OD	1	1	1	1	LP	Tn	2	0	19	0	0	0	1	0	1	0	5	4	0.7231	-0.7448	1	0.67329	0.32196
223	170200819	2017-01-27 18:45:37	Contusion	炮炸伤	1	1	0	0	0	13	3	13	Male	OS	3	2	0	1	HM	Tn	2	1	0	0	0	1	1	0	0	0	3	1	2.1134	0.7333	1	0.8922	0.67553
224	170200970	2017-01-29 21:38:07	Penetrating	树枝击伤	1	1	0	0	1	11	3	50	Male	OD	1	1	1	0	LP	T-1	1	1	17	0	0	1	1	1	0	0	4	3	2.2845	0.069	1	0.90758	0.51723
225	170201847	2017-02-08 23:21:06	Penetrating	铁屑溅伤	0	1	0	0	0	8	3	30	Male	OD	1	3	0	0	CM	Tn	2	0	15	0	0	0	1	0	0	0	5	2	0.7231	-0.7448	1	0.67329	0.32196
226	170201901	2017-02-06 15:57:38	Contusion	不明物砸伤	1	1	0	0	0	9	3	30	Female	OD	3	3	0	0	CF	Tn	2	0	0	0	0	1	1	0	0	0	5	2	2.1134	0.7333	1	0.8922	0.67553
227	170202968	2017-02-19 19:32:52	Rupture	砂轮片击伤	0	0	0	0	0	5	2	18	Male	OD	2	2	1	1	HM	Tn	2	1	15	0	0	0	1	0	0	0	3	1	-2.5632	-1.6251	0	0.07155	0.1645
228	170203234	2017-02-22 17:22:24	Penetrating	钢钉击伤	0	1	0	0	0	4	1	39	Male	OS	1	1	1	0	LP	Tn	2	1	12	0	1	0	1	0	0	0	4	3	-2.7812	-0.7448	1	0.05835	0.32196
229	170203243	2017-02-23 19:39:13	Rupture	木块碰伤	0	0	0	1	0	4	1	62	Male	OS	2	2	0	0	HM	Tn	2	1	14	0	1	0	1	0	0	0	5	3	-3.199	-0.9361	1	0.03921	0.28169
230	170203258	2017-02-22 11:30:28	Rupture	骑摩托车摔倒	1	1	0	0	1	9	3	21	Male	OS	2	2	0	0	HM	Tn	2	1	15	0	0	1	1	0	1	0	4	2	3.2505	1.008	1	0.96269	0.73263
231	170203889	2017-03-02 15:16:23	Penetrating	铁丝击伤	0	1	0	0	0	4	1	67	Male	OS	1	1	1	0	LP	Tn	2	1	6	0	1	0	1	0	0	0	5	4	-2.7812	-0.7448	1	0.05835	0.32196
232	170204474	2017-03-11 20:20:13	Contusion	铁块击伤	1	1	0	0	0	6	2	31	Male	OD	3	2	0	0	HM	Tn	2	1	0	0	0	0	1	0	0	0	4	2	0.7303	0.7333	1	0.67488	0.67553
233	170204519	2017-03-12 22:19:00	Contusion	烟花炸伤	1	1	0	0	0	19	4	57	Male	OD	3	2	0	3	HM	Tn	2	1	0	0	0	1	1	0	1	0	3	1	2.2544	0.7333	1	0.90503	0.67553
234	170204550	2017-03-14 04:26:12	Rupture	他人用拳头击伤	0	1	0	0	0	4	1	39	Female	OS	2	1	1	1	LP	Tn	2	1	18	0	1	0	1	0	0	0	3	2	-1.8153	0.1943	1	0.14	0.54841
235	170204661	2017-03-18 16:24:50	Rupture	抛光机砂轮片击伤	1	1	0	1	1	6	2	56	Male	OS	2	1	1	3	LP	T-1	1	0	15	2	0	1	0	0	0	0	0	-1	3.353	1.697	1	0.9662	0.84514
236	170205305	2017-04-07 17:02:33	Penetrating	钢钉扎伤	1	1	0	0	1	5	2	26	Male	OD	1	2	0	0	HM	Tn	2	1	15	0	0	0	1	0	0	0	4	2	0.9014	0.069	1	0.71125	0.51723

237	170205365	2017-04-09 17:40:09	Contusion	钢丝弹伤	1	0	0	1	0	19	4	70	Male	OD	3	3	0	0	CF	Tn	2	0	0	0	0	0	1	0	0	0	5	2	0.8707	-0.3971	1	0.70489	0.40202
238	170205376	2017-04-09 22:51:26	Penetrating	胶块碰伤	0	1	0	0	0	4	1	50	Male	OS	1	1	1	0	LP	Tn	2	1	5	0	1	0	1	0	1	0	4	3	-2.7812	-0.7448	1	0.05835	0.32196
239	170205673	2017-04-12 12:13:30	Rupture	拖拉机方向盘击伤	0	0	0	0	0	10	3	58	Male	OD	2	2	0	3	HM	Tn	2	1	15	0	1	0	1	0	0	0	5	3	-1.1802	-1.6251	0	0.23503	0.1645
240	170205802	2017-04-14 20:29:32	Penetrating	他人用不明物击伤	1	0	1	0	1	9	3	38	Male	OS	1	1	0	3	NLP	Tn	2	1	22	0	0	0	1	0	0	0	4	3	2.6973	0.9246	1	0.93687	0.71598
241	170205807	2017-04-15 12:35:40	Penetrating	塑料棍击伤	0	1	0	0	0	4	1	6	Male	OD	1	2	1	1	HM	Tn	2	1	14	0	1	0	1	0	0	0	5	3	-2.7812	-0.7448	1	0.05835	0.32196
242	170206290	2017-03-21 12:06:10	Rupture	扳手击伤	1	1	0	1	1	4	1	52	Male	OD	2	1	1	1	LP	Tn	2	0	30	3	1	0	1	0	0	0	2	1	1.2317	1.697	1	0.77411	0.84514
243	170206457	2017-03-27 09:46:33	Contusion	镰铲柄击伤	1	1	0	1	0	14	3	66	Male	OD	3	3	0	0	CF	Tn	2	0	0	0	0	1	1	0	0	0	3	0	3.5989	1.4223	1	0.97338	0.8057
244	170206951	2017-04-03 16:47:13	Contusion	木棍碰伤	1	1	0	0	0	14	3	14	Male	OS	3	2	1	1	HM	Tn	2	1	0	0	0	1	1	0	0	0	3	1	2.1134	0.7333	1	0.8922	0.67553
245	170207095	2017-04-20 15:05:26	Contusion	他人用弹弓击伤	1	0	0	0	1	25	4	11	Male	OS	3	1	0	0	LP	14.0	2	1	0	0	0	0	1	0	0	0	3	2	0.9465	-0.2723	1	0.72041	0.43233
246	170207432	2017-04-22 10:37:28	Contusion	木块崩伤	1	1	0	0	1	4	1	68	Male	OD	3	3	1	0	CF	Tn	2	1	0	1	1	0	1	0	0	0	5	2	0.1703	1.547	1	0.54248	0.82448
247	170207523	2017-04-23 18:28:37	Penetrating	钢丝扎伤	0	0	1	1	1	5	2	30	Male	OD	1	3	0	0	0.01	Tn	2	1	3	0	0	1	1	0	0	0	6	3	2.7998	1.6136	1	0.94267	0.83391
248	170207876	2017-04-30 02:26:02	Rupture	骑电动车摔伤	0	1	0	1	1	4	1	56	Male	OS	2	1	0	1	LP	Tn	2	0	23	3	1	0	1	1	0	0	3	2	1.2317	1.697	1	0.77411	0.84514
249	170208040	2017-05-05 21:41:47	Rupture	他人拳头打伤	1	1	0	1	1	12	3	50	Male	OS	2	1	0	2	LP	Tn	2	1	15	1	0	1	1	0	0	0	3	2	4.736	1.697	1	0.9913	0.84514
250	170208059	2017-05-07 11:35:44	Rupture	自行摔伤	0	1	0	0	0	11	3	47	Male	OS	2	2	0	1	HM	T-1	1	1	25	0	0	0	1	1	0	0	5	3	1.6891	0.1943	1	0.84411	0.54841
251	170208149	2017-05-02 15:04:24	Rupture	焦炭块击伤	0	0	0	1	0	3	1	70	Male	OS	2	2	1	1	HM	Tn	2	1	4	0	1	0	1	0	0	0	5	3	-3.199	-0.9361	1	0.03921	0.28169
252	170209285	2017-05-19 00:08:05	Rupture	骑摩托与拖拉机车斗相	1	1	0	0	1	10	3	17	Male	OD	2	1	0	1	LP	Tn	2	1	35	0	0	1	1	0	0	0	3	2	3.2505	1.008	1	0.96269	0.73263
253	170209315	2017-05-19 20:32:43	Contusion	他人用骨头投伤	0	0	0	0	0	14	3	37	Male	OS	3	3	1	0	CF	10.7	1	1	0	0	1	0	1	0	0	0	6	3	-0.7559	-1.0861	0	0.31954	0.25236
254	170209647	2017-05-23 15:51:43	Penetrating	钢筋击伤	1	1	0	1	1	9	3	43	Male	OD	1	2	0	0	HM	Tn	2	0	8	1	0	0	0	0	0	0	0	-2	3.77	0.758	1	0.97747	0.68091
255	170209756	2017-05-25 14:42:16	Rupture	骑电动车摔伤	1	1	0	0	0	9	3	53	Male	OD	2	1	1	4	LP	T-1	1	1	10	0	0	1	1	0	0	0	3	2	1.6891	0.1943	1	0.84411	0.54841
256	170209839	2017-05-28 10:40:37	Rupture	砂轮击伤	1	1	0	1	1	10	3	31	Female	OS	2	1	1	4	LP	T-2	1	1	15	2	0	1	1	1	0	0	2	1	4.736	1.697	1	0.9913	0.84514
257	170209871	2017-05-30 22:03:47	Penetrating	剪刀扎伤	0	1	0	0	0	10	3	10	Female	OD	1	2	1	0	HM	Tn	2	1	8	0	0	0	1	0	1	0	4	2	0.7231	-0.7448	1	0.67329	0.32196
258	170209883	2017-06-01 04:31:25	Rupture	他人用石头打伤	0	1	0	0	0	9	3	28	Male	OS	2	1	1	1	NLP	Tn	2	1	25	0	0	0	1	0	1	0	2	1	1.6891	0.1943	1	0.84411	0.54841
259	170210078	2017-06-02 15:03:15	Rupture	手指戳伤	1	1	0	0	0	5	2	20	Female	OS	2	2	0	1	HM	T-1	1	0	15	0	0	0	1	0	0	0	4	2	0.3061	0.1943	1	0.57593	0.54841
260	170210123	2017-06-04 14:42:48	Rupture	摇摆儿击伤	1	1	0	1	1	11	3	32	Male	OS	2	0	0	0	NLP	T-1	1	1	15	2	0	1	0	0	0	0	0	0	4.736	1.697	1	0.9913	0.84514
261	170210383	2017-06-16 16:46:18	Contusion	铝丝击伤	1	0	0	0	1	9	3	58	Male	OD	3	2	1	0	HM	Tn	2	1	0	0	0	0	1	0	0	0	4	2	0.8054	-0.2723	1	0.69114	0.43233
262	170210809	2017-06-27 17:54:20	Rupture	骑电动车摔伤	0	1	0	0	0	3	1	49	Male	OS	2	1	1	0	LP	T+1	3	1	14	0	1	0	1	0	0	0	2	1	-1.8153	0.1943	1	0.14	0.54841
263	170210875	2017-06-09 00:06:31	Rupture	石块碰伤	0	0	0	0	0	14	3	49	Male	OD	2	1	1	1	LP	T+1	3	1	25	0	1	0	1	0	0	0	5	4	-1.1802	-1.6251	0	0.23503	0.1645
264	170211484	2017-06-26 20:34:30	Penetrating	剪刀划伤	1	1	0	0	0	10	3	7	Male	OD	1	2	1	0	HM	Tn	2	0	11	0	0	0	1	0	0	0	4	2	0.7231	-0.7448	1	0.67329	0.32196
265	170211589	2017-06-28 16:28:16	Rupture	铁棍击伤	1	1	0	1	1	6	2	48	Male	OS	2	0	1	1	NLP	T-1	1	1	15	2	0	1	0	0	0	0	0	0	3.353	1.697	1	0.9662	0.84514
266	170211593	2017-06-30 07:05:17	Rupture	摔倒后相框碰伤	1	1	0	0	0	7	2	51	Male	OD	2	2	0	0	HM	Tn	2	0	20	0	0	0	1	0	0	0	5	3	0.3061	0.1943	1	0.57593	0.54841
267	170211758	2017-06-30 10:04:06	Perforating	铁片击伤	1	1	0	0	0	11	3	61	Male	OS	4	1	0	0	LP	Tn	2	1	9	0	0	1	1	0	0	0	5	4	4.2424	1.5081	1	0.98583	0.81878
268	170211784	2017-08-11 18:55:06	Rupture	原因不明的接触钝器	1	1	0	1	1	7	2	33	Male	OD	2	0	1	3	NLP	T-1	1	1	30	1	0	1	1	0	1	0	3	3	1.8674	1.008	1	0.86616	0.73263
269	170211787	2017-08-11 20:53:43	Penetrating	钢管弹伤	1	1	0	0	0	11	3	48	Male	OS	1	2	1	1	HM	Tn	2	1	17	0	0	0	1	0	1	0	4	2	0.7231	-0.7448	1	0.67329	0.32196
270	170211811	2017-07-01 17:56:15	Rupture	砖块打伤	1	1	0	0	1	7	2	62	Male	OD	2	1	0	1	LP	18.8	2	0	15	0	0	1	1	0	1	0	3	2	1.8674	1.008	1	0.86616	0.73263
271	170212344	2017-07-05 17:54:09	Rupture	骑电动车与叉车相撞	1	1	0	0	1	12	3	65	Male	OS	2	2	1	0	HM	Tn	2	1	5	0	0	0	1	0	0	0	4	2	3.2505	1.008	1	0.96269	0.73263
272	170212805	2017-07-11 18:31:56	Rupture	铁管戳伤	0	0	0	0	0	11	3	45	Male	OS	2	2	0	0	HM	Tn	2	1	11	0	1	0	1	0	0	0	5	3	-1.1802	-1.6251	0	0.23503	0.1645
273	170213098	2017-07-16 13:14:40	Contusion	原因不明的接触钝器	0	1	0	0	0	3	1	60	Female	OD	3	2	1	0	HM	Tn	2	1	0	0	1	0	1	0	0	0	5	3	-1.391	0.7333	1	0.19925	0.67553
274	170213188	2017-07-17 16:22:26	Rupture	车祸	1	1	0	1	0	9	3	27	Male	OS	2	3	1	0	CF	Tn	2	1	15	0	0	0	1	0	0	0	6	3	3.1747	0.8833	1	0.95987	0.7075
275	170213281	2017-07-19 00:44:28	Contusion	高压管击伤	1	1	0	0	0	9	3	27	Male	OD	3	1	0	0	LP	Tn	2	1	0	0	0	1	1	0	0	0	2	1	2.1134	0.7333	1	0.8922	0.67553
276	170213299	2017-07-18 10:05:16	Penetrating	合金片扎伤	0	1	0	0	0	5	2	25	Male	OS	1	1	1	0	LP	T-1	1	1	15	0	0	0	1	0	0	0	5	4	-0.6599	-0.7448	1	0.34076	0.32196
277	170213375	2017-07-18 14:17:38	Penetrating	飞起的螺丝击伤	1	1	0	0	0	12	3	46	Female	OD	1	1	1	0	LP	T-1	1	1	20	0	0	0	1	0	0	0	4	3	0.7231	-0.7448	1	0.67329	0.32196
278	170213509	2017-07-21 03:43:41	Contusion	钢丝绳击伤	1	1	0	0	1	14	3	42	Male	OS	3	3	0	0	CF	Tn	2	0	0	0	0	0	1	0	0	0	5	2	3.6747	1.547	1	0.97527	0.82448
279	170213569	2017-07-22 13:36:24	Penetrating	钢筋击伤	1	1	0	1	1	6	2	46	Male	OS	1	1	1	0	LP	40.0	3	1	5	2	0	1	0	0	0	0	0	-1	2.387	0.758	1	0.91583	0.68091
280	170214001	2017-07-28 17:04:00	Penetrating	金属丝扎伤	0	0	0	0	0	12	3	47	Male	OD	1	2	1	1	HM	13.2	2	0	3	0	1	0	1	0	0	0	6	4	-2.1461	-2.5641	0	0.10469	0.07148
281	170214577	2017-08-04 12:49:06	Perforating	铁屑	1	1	0	0	0	12	3	37	Male	OS	4	4	0	0	0.12	Tn	2	0	6	0	0	1	1	0	0	0	5	1	4.2424	1.5081	1	0.98583	0.81878
282	170214979	2017-08-08 17:27:57	Contusion	磨光机片崩伤	1	1	0	1	1	4	1	39	Male	OD	3	2	0	1	HM	T+1	3	1	0	2	1	1	1	0	0	0	4	2	1.6559	2.236	1	0.83969	0.90344
283	170215336	2017-08-14 14:47:52	Rupture	不明物击伤	0	0	1	1	1	4	1	64	Male	OS	2	1	1	1	LP	T-1	1	0	15	0	1	1	1	0	0	0	3	2	1.6445	2.5526	1	0.83815	0.92775
284	170215819	2017-08-25 14:50:24	Penetrating	剪刀扎伤	1	1	0	0	1	4	1	7	Male	OD	1	2	1	1	HM	Tn	2	1	6	0	1	0	1	0	0	0	5	3	-1.2199	0.069	1	0.22795	0.51723
285	170216267	2017-08-26 16:23:19	Contusion	砂轮片击伤	1	1	0	0	1	10	3	33	Male	OD	3	2	1	1	HM	T+1	3	1	0	0	0	1	1	0	0	0	2	0	3.6747	1.547	1	0.97527	0.82448
286	170216889	2017-09-07 11:25:53	Contusion	塑料管打伤	0	0	0	1	1	3	1	55	Male	OS	3	1	1	1	LP	T+1	3	0	0	0	1	0	1	0	0	0	3	2	-1.2134	0.4167	1	0.22911	0.60268
287	170217332	2017-09-16 11:35:46	Rupture	钢筋击伤	0	0	0	1	0	3	1	59	Male	OS	2	2	1	0	HM	9.0	1	1	19	0	1	0	1	0	0	0	5	3	-3.199	-0.9361	1	0.03921	0.28169
288	170217521	2017-09-21 09:39:54	Penetrating	钉子击伤	0	0	0	0	0	10	3	17	Male	OD	1	2	1	0	HM	Tn	2	1	3	0	1	0	1	0	0	0	5	3	-2.1461	-2.5641	0	0.10469	0.07148
289	170217939	2017-09-30 18:24:02	Rupture	三轮车碰伤	0	0	0	0	0	12	3	60	Male	OD	2	1	0	3	LP	Tn	2	1	12	0	1	0	1	0	0	0	5	4	-1.1802	-1.6251	0	0.23503	0.1645
290	170217945	2017-10-01 14:12:57	Contusion	木块击伤	1	0	0	1	0	9	3	60	Female	OS	3	1	1	1	LP	9.0	1	0	0	0	0	0	1	0	0	0	3	2	0.7297	-0.3971	1	0.67473	0.40202
291	170217961	2017-10-04 22:35:07	Rupture	钢筋碰伤	0	0	0	0	0	14	3	43	Male	OS	2	1	1	0	LP	Tn	2	0	15	0	1	0	1	0	0	0	4	3	-1.1802	-1.6251	0	0.23503	0.1645
292	170218192	2017-09-30 12:11:29	Rupture	磨光机片崩伤	1	0	0	1	0	9	3	54	Male	OD	2	2	0	0	HM	Tn	2	1	9	0	0	0	1	1	0	0	3	1	0.3054	-0.9361	1	0.57576	0.28169
293	170218444	2017-10-09 17:22:01	Penetrating	石子打伤	0	0	0	0	0	10	3	51	Male	OD	1	2	0	1	HM	Tn	2	0	5	0	1	0	1	0	0	0	5	3	-2.1461	-2.5641	0	0.10469	0.07148
294	170219182	2017-10-18 14:33:09	Contusion	铁丝击伤	0	0	0	1	1	4	1	62	Male	OS	3	3	1	1	CF	T+2	3	0	0	0	1	0	1	0	0	0	6	3	-1.2134	0.4167	1	0.22911	0.60268
295	170219253	2017-10-18 17:04:58	Rupture	摔伤	1	0	0	1	0	9	3	51	Male	OD	2	0	1	1	NLP	T-1	1	1	20	0	0	1	1	0	0	0	2	2	0.3054	-0.9361	1	0.57576	0.28169
296	170219315	2017-10-21 00:28:15	Penetrating	铁丝击伤	0	0	0	0	0	14	3	47	Male	OS	1	2	0	0	HM	Tn	2	1	4	0	1	0	1	0	0	0	6	4	-2.1461	-2.5641	0	0.10469	0.07148
297	170219429	2017-10-25 19:25:26	Contusion	他人用不明物体击伤	1	0	0	0	1	9	3	55	Male	OD	3	1	1	1	LP	10.2	1	0	0	0	0	0	1	0	0	0	3	2	0.8054	-0.2723	1	0.69114	0.43233
298	170219786	2017-10-28 12:08:42	Rupture	铁管击伤	0	0	0	0	0	10	3	41	Male	OD	2	2	1	4	HM	6.0	1	1	20	0	1	0	1	0	0	0	5	3	-1.1802	-1.6251	0	0.23503	0.1645
299	170219819	2017-10-31 11:51:05	Contusion	木柴击伤	0	0	0	1	0	4	1	72	Male	OD	3	4	0	0	0.20	20.3	2	1	0	0	1	0	1	0	0	0	6	2	-2.7747	-0.3971	1	0.05871	0.40202
300	170220440	2017-11-06 17:26:38	Penetrating	铁器崩伤	0	0	0	0	0	10	3	32	Male	OS	1	2	1	1	HM	Tn	2	1	13	0	1	0	1	0	0	0	5	3	-2.1461	-2.5641	0	0.10469	0.07148
301	140135056	2014-01-20 22:10:06	Rupture	钢筋击伤	0	1	0	1	1	2	1	24	Male	OD	2	1	1	1	LP	T-2	1	0	15	2	1	0	1	0	0	0	3	2	1.2317	1.697	1	0.77411	0.84514
302	140147695	2014-08-26 11:09:14	Rupture	钢筋碰伤	1	1	0	0	0	5	2	59	Male	OS	2	1	1	0	LP	Tn	2	1	25	0	0	1	1	0	0	0	3	2	0.3061	0.1943	1	0.57593	0.54841
303	140149545	2014-10-04 14:21:20	Rupture	他人拳头击伤	0	1	0	0	1	4	1	27	Male	OD	2	1	0	1	LP	T-1	1	0	21	1	1	0	1	0	0	0	3	2	-0.2539	1.008	1	0.43686	0.73263
304	160180856	2016-03-08 23:48:48	Rupture	他人打伤	1	1	0	0	0	7	2	32	Male	OD	2	1	1	0	LP	Tn	2	1	30	0	0	0	1	0	0	0	3	2	0.3061	0.1943	1	0.57593	0.54841
305	160192017	2016-08-28 02:58:52	Rupture	车祸	1	1	0	0	0	6	2	51	Male	OD	2	1	1	0	LP	Tn	2	1	27	0	0	0	1	0	0	0	4	3	0.3061	0.1943	1	0.57593	0.54841
